# How Staff, Patients, Residents and Families Perceive Mobilisation Alarms for Preventing Falls in Hospital and Aged Care: A Scoping Review

**DOI:** 10.1111/jocn.70486

**Published:** 2026-08-05

**Authors:** Kelly Stephen, Dai Pu, Jennifer Weller‐Newton, Ravi Manohar, Terry P. Haines

**Affiliations:** ^1^ School of Primary and Allied Health Care, Faculty of Medicine, Nursing and Health Sciences Monash University Frankston Victoria Australia; ^2^ Eastern Health Box Hill Victoria Australia; ^3^ Faculty of Medicine and Health Sciences The University of Melbourne Melbourne Victoria Australia; ^4^ Faculty of Health, School of Nursing & Midwifery University of Canberra Canberra ACT Australia

## Abstract

**Aim:**

To compare and contrast evidence investigating the use of mobilisation alarms in hospitals and residential aged care, exploring: (1) why and how mobilisation alarms are used; (2) attitudes of staff, patients, residents and family members towards mobilisation alarm use and effectiveness.

**Methods:**

A scoping review was conducted following JBI methodology. Two independent reviewers screened studies against predefined inclusion criteria, and one reviewer extracted data. Quantitative data were predominantly converted into textual descriptions to enable narrative and thematic syntheses.

**Data Sources:**

Eight databases were searched in July 2025 with no date or language restrictions.

**Results:**

Fifty‐eight studies were included, with 81% hospital‐based and primarily examining staff perspectives. Alarm types included traditional pressure‐sensitive pads, more advanced integrated bed alarms and wearable alarms. Alarms were frequently used; however, authors cautioned about ongoing high investment in traditional alarms. Themes that shaped attitudes towards alarms were effectiveness, device knowledge, workload impact, liability concerns and ethical tensions.

**Conclusion:**

Overuse and limited effectiveness of traditional pressure‐sensitive alarms have shaped negative attitudes towards them. These factors, alongside emerging innovative alarm technologies, warrant future research efforts to explore alternative fall prevention strategies that are effective and acceptable to all stakeholders.

**Impact:**

Evidence shows that the effectiveness of alarms in preventing falls is uncertain, but it was unclear how often and in what ways alarms were being used across hospital and residential aged care. This scoping review identified that research is heavily skewed towards hospitals and nurse perspectives of alarms, with limited attention to residential aged care and patient, resident, family or non‐nurse perspectives. Aged care initiated alarm disinvestment earlier than hospitals, but its translation to other facilities or sustainment is unknown. Such insights may assist hospital staff who are beginning their own disinvestment journey.

**Reporting Method:**

PRISMA‐ScR guidelines.

**Patient or Public Contribution:**

No Patient or Public Contribution.

## What Does This Paper Contribute to the Wider Global Clinical Community?

- This scoping review identified that the main types of mobilisation alarms in use across hospitals and residential aged care are traditional pressure‐sensitive pads, more advanced integrated bed alarms, and wearable alarms.
- Attitudes towards mobilisation alarms are shaped by perceptions of their effectiveness, impact on workload, concerns about liability and underlying ethical tensions. Nurse perspectives on alarms in hospital settings are well studied; however, there is limited evidence about experiences in residential aged care or the views of patients, residents and their families.
- Residential aged care led early efforts in alarm disinvestment but long‐term sustainment and broader adoption remain unknown. Understanding how aged care managed this transition can offer valuable lessons for hospitals starting their own disinvestment journeys.

## 1 Introduction

Falls are a common adverse event occurring in hospital (Dykes et al. 2023) and residential aged care (RAC) (Wabe et al. 2022). Mobilisation alarms are devices designed to detect movement and alert healthcare staff when a person at risk of falling attempts to mobilise unassisted. Positioned in or around a person's immediate environment, these systems intend, at least in theory, to prevent falls by alerting staff to intervene before the person attempts to leave their bed or chair. However, the practical effectiveness of these alarms depends on the timeliness of staff response once triggered. Alarms come with a variety of notification methods (bedside audio alerts, visual alerts via the nurse call system, lights outside the rooms or pager systems) and trigger mechanisms (pressure‐sensitive pads integrated into beds (Clodfelter 2025), socks (Cumming et al. 2008) or portably placed on beds, chairs or the floor beside the bed (Wong Shee et al. 2014), pull‐string activated alarms attached to the person (Bressler et al. 2011), accelerometer‐based wearable devices (Dollard et al. 2022) and infrared motion detectors) (Vandenberg et al. 2017). Recent systematic reviews indicate alarms may not prevent falls (Cortés et al. 2021; Morris et al. 2022), albeit most research in these reviews involved use of pressure‐sensitive pad trigger technologies.

Mobilisation alarms appear to be frequently used for falls prevention in hospital and RAC settings despite the limited evidence supporting their use for this purpose. Early randomised controlled trials (RCT) and non‐randomised trials from the 1990s onwards appeared to be supportive (Tideiksaar et al. 1993), though later RCTs from the 2010s onwards did not reproduce the same results (Shorr et al. 2012). There is evidence that some RAC settings began disinvesting from mobilisation alarms in publications as early as 2011 (Bressler et al. 2011), while in hospitals the first publication to report on disinvesting from alarms appeared as a pilot in 2021 (Brusco et al. 2021). This presents a complex picture of heterogeneity in currently applied models of falls prevention care across both settings, and raises the question as to why this might be the case. It is unclear the extent of literature that explores whether those who use alarms find them helpful for preventing falls or whether usage patterns are similar across hospitals and RAC. One previous review explored alarm effectiveness for falls prevention in RAC and identified facilitators and barriers (Mileski et al. 2019). It noted that perceptions of alarms have improved with newer alarm technologies, and emphasised the importance of educating staff on proper alarm use. However, it did not examine other patterns of alarm research over time, reasons for alarm use, or how stakeholder attitudes might explain any differences between hospital and RAC settings. The current review builds on this work by providing a more nuanced understanding of how the evidence has developed over time and explicitly compares hospital and RAC settings to help direct future recommendations regarding mobilisation alarm use in falls prevention.

## 2 Aims

The objective of this research was to describe, compare and contrast current evidence investigating the use of alarms in hospitals and RAC, with specific regard to:

1. Why and how are mobilisation alarms used?
2. What are the attitudes among staff, patients, residents and family members towards the use and effectiveness of mobilisation alarms?

## 3 Methods

### 3.1 Design

A systematic review was initially planned (CRD420251051480) (Centre for Reviews and Dissemination 2025), however, the breadth and heterogeneity of included studies required a methodological pivot to a scoping review. A scoping review approach was deemed most appropriate as its core purposes are to map a broad body of literature, clarify concepts and identify evidence gaps regarding mobilisation alarm use across both hospital and RAC sectors (Pollock et al. 2021; Peters et al. 2024). The primary protocol deviation was the decision to omit the planned quality appraisal as we were not seeking to estimate a pooled treatment effect or make statements that were intended to be representative of a broader population. A second deviation was limiting the breadth of this scoping review to report only on mobilisation alarms other than video monitoring technology, as it was considered that video monitoring is used for a range of purposes much broader than just falls and is implemented through processes distinct from other mobilisation alarms (e.g., they commonly involve the introduction of video monitoring technicians) which is likely to generate a distinct set of issues and responses related to our research questions. This scoping review followed the Joanna Briggs Institute's (JBI) nine stages for scoping reviews (Peters et al. 2024) and is reported according to PRISMA Extension for Scoping Reviews (PRISMA‐ScR) guidance (Tricco et al. 2018).

### 3.2 Inclusion Criteria

The a priori protocol was adhered to regarding eligibility criteria, searching, selecting and extracting evidence. Eligibility criteria used a Participant, Concept and Context approach (Table S1). All study designs and grey literature were eligible, with no date or language restrictions. Participants included staff, patients, residents or families. The concept was evidence pertaining to how and why alarms were used and attitudes towards their use.

### 3.3 Search Methods

A preliminary search was conducted in JBI evidence synthesis and Cochrane Database for Systematic reviews in May 2025 and found no existing reviews that specifically synthesised alarm implementation and reported attitudes towards them. Additional searching in Google Scholar retrieved one review which did not fully address our research questions (Mileski et al. 2019).

A further limited search of Medline and CINAHL was undertaken to find studies on the topic and identified keywords and index terms to include in the full search strategy. The final search strategy was devised in consultation with an academic librarian (Table S2). Databases were searched in July 2025 using key words and index terms, adapted for each database. Databases included Ovid (Medline, Embase, Emcare and PsychINFO), EBSCO (CINAHL and Ageline), Scopus and Clinical Trials registry (for linked qualitative process evaluations). Indexed grey literature (such as conference abstracts, dissertations and book chapters) within these academic databases was screened for eligibility. To capture additional grey literature, Google Scholar was searched using key term combinations and the first 100 results were screened for eligibility. Studies were imported into Endnote 21 and duplicates removed prior to exporting into Covidence systematic review software. Reference lists of included studies and retrieved systematic reviews were screened for additional studies. One protocol author was contacted regarding a planned study, though data was not yet available.

### 3.4 Study Selection

Two independent reviewers (KS & RM) screened titles and abstracts in Covidence and assessed full texts of potentially relevant studies against the inclusion criteria. Reasons for exclusion of full text studies are reported in the PRISMA flow chart (Figure 1). Conflicts at both title/abstract and full‐text screening stages were resolved by a third reviewer (DP).

**FIGURE 1:**
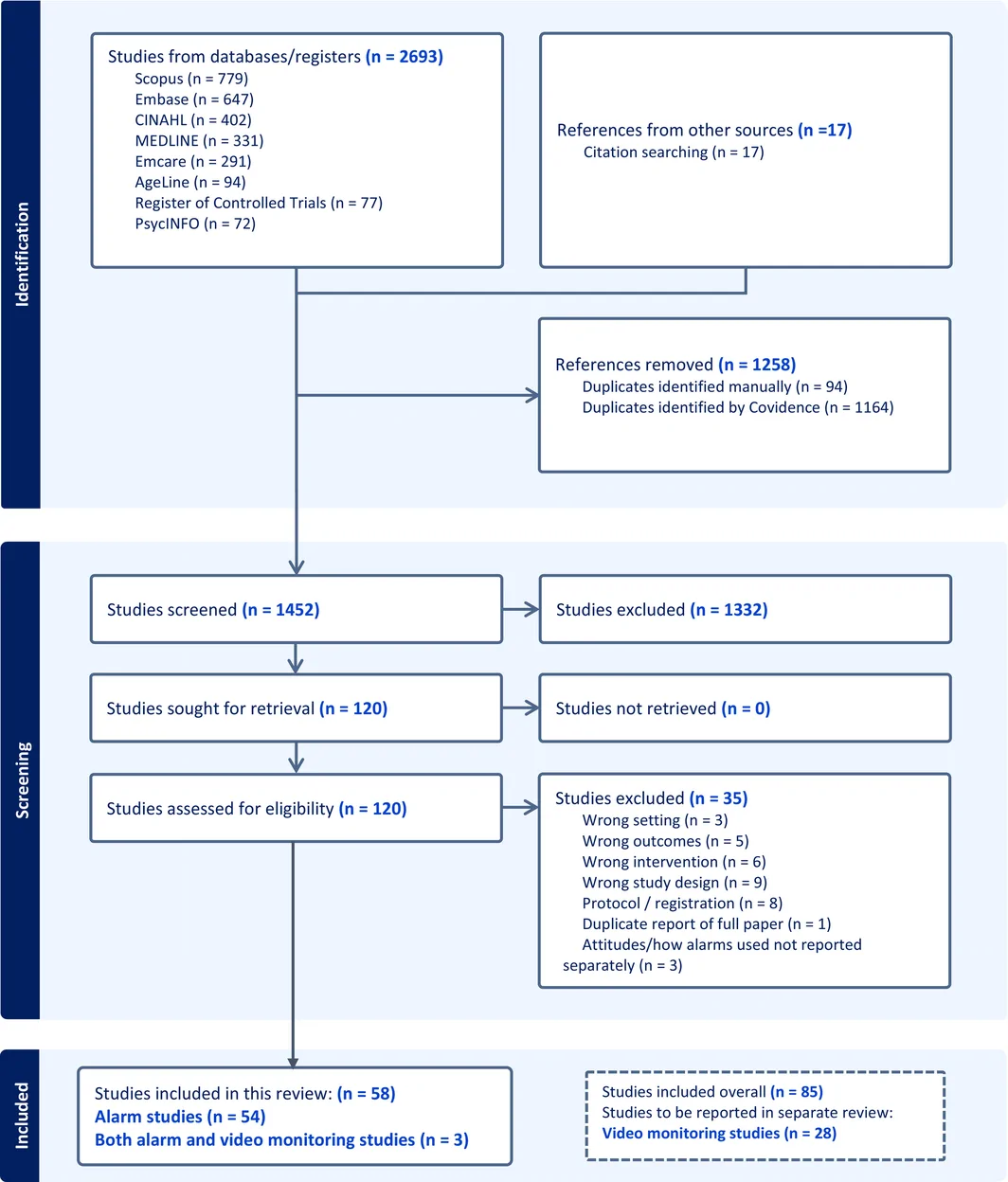
PRISMA diagram outlining data screening. [Colour figure can be viewed at wileyonlinelibrary.com]

### 3.5 Data Charting

A data charting template was developed a priori in Microsoft Excel and used to extract data from any section of the articles. Data items included: author; year; country/setting; alarm type; methodology; sample characteristics and key findings. Although formal critical appraisal is not required in scoping reviews, we retained fields in the charting form to record basic study characteristics, methodological design details and author‐reported limitations to provide contextual information about the overall robustness of the evidence base and help inform future research agendas. One reviewer (KS) independently extracted data following a team planning meeting about how data would be extracted using the agreed upon template. Intermittent checks were completed with other authors. Piloting the template on five studies confirmed its adequacy, with no major alterations required.

### 3.6 Data Analysis and Presentation

Data related to study characteristics (design, year of publication, setting, country, alarm type and study focus) were presented descriptively. A convergent integrated approach to synthesis combined quantitative and qualitative evidence. Quantitative data were transformed to textual descriptions to allow integration with qualitative data, though some numbers were not eliminated to preserve precision and allow readers to understand the strength of evidence.

A narrative synthesis approach (without robustness assessment) was used to tell the story of why and how alarms are used via developing a theory, describing patterns across the studies and exploring relationships in the data (Popay et al. 2006). A thematic synthesis approach was used for a deeper exploration of attitudes towards the use and effectiveness of alarms (Thomas and Harden 2008). The primary reviewer (KS) performed inductive line‐by‐line coding of each study using colour‐coded matrices in Microsoft Word, synthesising codes into descriptive categories by identifying similarities and differences in meaning. To ensure rigour and verification, these categories were then subjected to further synthesis and interpretation during regular team meetings with co‐authors until consensus was reached to produce the final analytical themes.

### 3.7 Rigour

Multiple approaches were adopted to optimise transparency and trustworthiness. The protocol was registered with PROSPERO (given the original plan for a systematic review) and adheres to the PRISMA Extension for Scoping Reviews (PRISMA‐Scr) reporting guidelines. Credibility was established by the lead researcher's prolonged engagement with the data over an extended period. The research team's diverse expertise and backgrounds, reinforced by regular meetings to discuss the review and findings, facilitated confirmability. Reflexive journaling and an audit trail were maintained by the lead researcher. This documented key decisions, personal reflections and supervisor feedback across all stages of the review (planning, searching, screening and synthesis). Inclusion of both peer‐reviewed and grey literature supported representation of a range of evidence.

## 4 Findings

### 4.1 Study Selection

Fifty‐eight studies were included in this review (Figure 1). The initial search yielded 2693 studies. Title and abstract screening reduced the number for full text review to 120. Application of the inclusion and exclusion criteria resulted in 58 studies selected for review.

### 4.2 Characteristics of Included Studies

Characteristics of included alarm studies are outlined in Tables 1 and 2—for a summary of studies' aims and key findings refer to Table S3. The majority of studies (81%) were hospital‐based. Most studies originated from the United States (U.S.), with Australia contributing the next largest share. Research predominantly focussed on portable pressure pad alarms. Twenty studies (35%) evaluated alarms within the broader context of falls prevention. Perspectives of a newly implemented alarm were explored in 17 studies (29%), whilst the remaining studies evaluated alarms already embedded in routine care. Studies primarily examined staff perspectives on alarms, with a smaller proportion including patient, resident or family perspectives. Quantitative studies tended to reveal positive perspectives, whereas qualitative studies reflected a mix of positive and negative perspectives. As attitudes towards alarm effectiveness changed over time, alarm prevalence and disinvestment studies started to emerge.

**TABLE 1:** Study characteristics mapping.

Author	Year	Setting		Study design			Country	Study focus				Alarm type			
		Hospital	RAC	Quantitative	Qualitative	Mixed method		Staff attitude	Patient Family attitude	Alarm usage	Disinvestment	Pressure pad	Integrated	Wearable	Not specified
Anderson and Breda (2025)	2025	🗸		🗸			United States	🗸					🗸		
Balaguera et al. (2017)	2017	🗸				🗸	United States	🗸					🗸		
Barker et al. (2017)	2017	🗸				🗸	Australia	🗸				🗸			
Bressler et al. (2011)	2011		🗸	🗸			United States	🗸			🗸	🗸	🗸		
Brusco et al. (2021)	2021	🗸		🗸			Australia			🗸	🗸	🗸			
Clodfelter (2025)	2025	🗸		🗸			United States	🗸					🗸		
Considine et al. (2023)	2023	🗸			🗸		Australia	🗸				🗸			
Crogan and Dupler (2014)	2014		🗸	🗸			United States	🗸			🗸				🗸
Diduszyn et al. (2008)	2008	🗸		🗸			United States	🗸				🗸			
Dollard et al. (2022)	2022	🗸				🗸	Australia		🗸					🗸	
Fehlberg et al. (2018)	2018	🗸		🗸			United States			🗸					🗸
Fehlberg et al. (2020)	2020	🗸			🗸		United States	🗸				🗸			
Ferrari et al. (2012)	2012	🗸		🗸			United States	🗸	🗸					🗸	
Fisher et al. (2014)	2014	🗸				🗸	United States	🗸				🗸			
Gordon et al. (2015)	2015		🗸	🗸			United States	🗸			🗸				🗸
Gray (2013)	2013	🗸				🗸	United States		🗸						🗸
Grealish et al. (2019)	2019	🗸		🗸			Australia			🗸					🗸
Hall et al. (2017)	2017		🗸				United Kingdom	🗸	🗸			🗸			
Hartmann et al. (2021)	2021		🗸		🗸		United States	🗸			🗸	🗸			
Hill et al. (2022)	2022	🗸		🗸			United States			🗸		🗸			
Hoke and Zekany (2020)	2020	🗸			🗸		United States	🗸				🗸			
Hubbartt et al. (2011)	2011	🗸			🗸		United States	🗸				🗸			
Iio et al. (2016)	2016		🗸			🗸	Japan	🗸	🗸			🗸			
Jagella et al. (1992)	1992		🗸	🗸			United States	🗸	🗸			🗸			
Johnston and Magnan (2019)	2019	🗸		🗸			United States			🗸			🗸		
Jones et al. (2021)	2021	🗸		🗸			United States	🗸	🗸			🗸			
Kelly et al. (2002)	2002		🗸	🗸			United States	🗸	🗸					🗸	
King et al. (2018)	2018	🗸			🗸		United States	🗸				🗸			
King et al. (2021)	2021	🗸			🗸		United States		🗸			🗸			
Lopez et al. (2010)	2010	🗸				🗸	United States	🗸				🗸			
Matsuo et al. (2008)	2008	🗸				🗸	United States	🗸	🗸				🗸		
Mitchell et al. (2018)	2018	🗸		🗸			Australia			🗸		🗸			
Okumoto et al. (2020)	2020	🗸		🗸			Japan	🗸							🗸
Porter et al. (2018)	2018	🗸			🗸		United States	🗸				🗸			
Pu et al. (2025)	2026	🗸		🗸			Australia			🗸	🗸	🗸	🗸		
Radecki et al. (2018)	2018	🗸			🗸		United States		🗸			🗸			
Sarkies et al. (2019)	2019	🗸		🗸			Australia	🗸							🗸
Shever et al. (2011)	2011	🗸		🗸			United States			🗸					🗸
Shinmoto Torres et al. (2017)	2017	🗸		🗸			Australia		🗸					🗸	
Skolka et al. (2023)	2023	🗸		🗸			United States	🗸				🗸			
Staggs et al. (2020)	2020	🗸		🗸			United States			🗸					🗸
Stephen and Campbell (2023)	2023	🗸			🗸		Australia		🗸			🗸			
Stephen, Pu, Weller‐Newton, and Haines (2026)	2025	🗸			🗸		Australia	🗸				🗸	🗸		
Subermaniam et al. (2016)	2017	🗸		🗸			Malaysia	🗸				🗸			
Takase (2023)	2023	🗸		🗸			Japan			🗸		🗸			
Tideiksaar et al. (1993)	1993	🗸		🗸			United States	🗸	🗸			🗸			
Timmons et al. (2019)	2019	🗸			🗸		United Kingdom	🗸	🗸			🗸			
Turner et al. (2025)	2025	🗸				🗸	United States	🗸			🗸				🗸
Tzeng and Yin (2015)	2015	🗸		🗸			United States	🗸							🗸
Vandenberg et al. (2017)	2017		🗸		🗸		United States/Netherlands	🗸				🗸			
Vassallo et al. (2005)	2005	🗸		🗸			United Kingdom	🗸	🗸			🗸			
Wen et al. (2022)	2022	🗸		🗸			Taiwan	🗸				🗸	🗸		
Widder (1985)	1985	🗸		🗸			United States	🗸	🗸					🗸	
Wolf et al. (2013)	2013	🗸		🗸			Germany	🗸	🗸					🗸	
Wong Shee et al. (2014)	2014	🗸				🗸	Australia	🗸				🗸			
Yesmin et al. (2022)	2022	🗸				🗸	Canada	🗸					🗸		
Zwijsen et al. (2011)	2011		🗸			🗸	Netherlands	🗸	🗸			🗸			
Zwijsen et al. (2012)	2012		🗸		🗸		Netherlands	🗸				🗸			

**TABLE 2:** Overview of study characteristics.

Category	Subcategory	Hospital 47	RAC 11	Total 58
Alarm type	Pressure pad	34 (72%)	9 (82%)	43 (74%)
	Integrated in bed	11 (23%)	1 (9%)	12 (21%)
	Body worn	5 (11%)	1 (9%)	6 (10%)
Topic focus	Staff attitudes	23 (49%)	6 (55%)	29 (50%)
	Patient, resident, family attitudes	6 (13%)	0	6 (10%)
	Both staff & patient/resident attitudes	8 (17%)	5 (45%)	13 (22%)
	Alarm frequency	6 (13%)	1 (9%)	7 (12%)
	Disinvestment	3 (6%)	4 (36%)	7 (12%)
Year of publication	1976–1985	1 (2%)	0	1 (2%)
	1986–1995	1 (2%)	1 (9%)	2 (3%)
	1996–2005	1 (2%)	1 (9%)	2 (3%)
	2006–2015	11 (23%)	5 (45%)	16 (28%)
	2016–2026	32 (68%)	4 (36%)	36 (62%)
	2026	1 (2%)	0	1 (2%)
Country	United States	27 (57%)	7 (64%)	34 (59%)
	Australia	12 (26%)	0 (0%)	12 (21%)
	Asia	4 (9%)	1 (9%)	5 (9%)
	Europe	1 (2%)	3 (27%)	4 (7%)
	United Kingdom	2 (4%)	1 (9%)	3 (5%)
	Canada	1 (2%)	0	1 (2%)
Study design	Quantitative	26 (55%)	5 (45%)	31 (53%)
	Qualitative	11 (23%)	4 (36%)	15 (26%)
	Mixed methods	10 (21%)	2 (18%)	12 (21%)

### 4.3 How Alarms Are Used

#### 4.3.1 Models of Alarms Used

Alarm systems have changed over time. Early studies investigated pressure pads, but there were drawbacks associated with these devices (e.g., false alarms, faults, loose cords, pressure injury concerns, easily misplaced or avoided), and user error (e.g., forgetting to switch on, incorrect device positioning, ignoring alerts) (Grealish et al. 2019; Hill et al. 2022; Skolka et al. 2023; Takase 2023). Technological advancements enabled the creation of alternative alarms. Recent research has increasingly focused on integrated bed alarms, with only two studies (Hill et al. 2022; Skolka et al. 2023) continuing to promote traditional alarm use. No RCTs examined superiority of integrated bed alarms. However, findings from pre‐post and qualitative studies suggested integrated alarms had better specificity via its customised alert settings which reportedly improved staff responsiveness and reduced negative workload impacts such as alarm fatigue, distraction and excessive noise (Clodfelter 2025; Balaguera et al. 2017; Hoke and Zekany 2020; Wen et al. 2022; Yesmin et al. 2022). Again, user error was claimed to be associated with these alarms, such as incorrect setting selection and inadequate response times leading to educational campaigns targeting correct alarm settings and policies such as ‘no pass,’ where all nearby team members were expected to answer alerts (Johnston and Magnan 2019; Hoke and Zekany 2020; Clodfelter 2025).

Research on wearable alarms was minimal, represented by only six studies out of the 58 reviewed. Widder (Widder 1985) first evaluated the use of a thigh‐attached alarm in 1985, and more recently in 2022, Dollard et al. (2022) evaluated an alarm embedded in a vest. All studies, albeit small pilot studies, demonstrated high patient and staff acceptability. Despite wearable alarm research spanning decades, use of such alarms has not translated into standard practice in either hospitals or RAC.

#### 4.3.2 Frequency of Alarm Use

Hospital resource limitations, the ongoing issue of falls and their associated costs, and uncertainty regarding alarm effectiveness, prompted research surveying how frequently alarms were used (Mitchell et al. 2018; Shever et al. 2011; Staggs et al. 2020). In a survey of 140 acute nurse managers, alarms were identified as the most used fall prevention strategy, reported in 90% of the cases (Shever et al. 2011). Research across six large Australian health services estimated alarms consumed 11% of total fall prevention expenditure (Mitchell et al. 2018). A study across 57 U.S.‐based hospitals found alarms were used for 531 (36%) patients, with usage associated with unit ‘custom’ rather than patient‐specific fall risk factors (Staggs et al. 2020). These studies which sought to quantify how much alarms are being used, all warned about the ongoing high investment in hospital alarm use and called for consideration of alarm disinvestment. Whilst no formal alarm prevalence studies were found in RAC, researchers have reported high alarm use—between 21%–49% (Crogan and Dupler 2014) to 75% of residents (Bressler et al. 2011) prior to alarm disinvestment efforts.

#### 4.3.3 Alarm Disinvestment

Data related to alarm disinvestment in RAC entered the literature over a decade ago. Whilst researchers focused on the hospital setting have been discussing this over a similar period, except for Pu et al. (2025), they have not actually reported undertaking it at scale yet. Despite ongoing trial and adoption of alarms in many settings, several studies recommended reducing alarm use or, at a minimum, re‐evaluating current high‐use practices, given their limited effectiveness, perceived restraint and associated negative impacts (Barker et al. 2017; Mitchell et al. 2018; Radecki et al. 2018; Staggs et al. 2020).

Where disinvestment from mobilisation alarms has been undertaken within the RAC setting, it was with the view that alarms potentially dehumanised person‐centred care. There were concerns that staff were too dependent on alarms and waited for the alarm to alert them to the resident's needs. Alarm disinvestment aimed to improve overall care delivery and promote dignity through a more comfortable environment, facilitating staff to be proactive and attuned to residents' needs by developing a more holistic knowledge of them (Bressler et al. 2011; Crogan and Dupler 2014; Gordon et al. 2015; Hartmann et al. 2021). In the hospital setting, staff prioritised alarm use to enhance safety, often at the expense of patients' freedom of movement and privacy. Only one RAC study included staff comments that similarly prioritised safety over autonomy (Hall et al. 2017), potentially explaining why disinvestment was more readily embraced in RAC than in hospitals.

The first hospital study of alarm disinvestment was a small Australian pilot (Brusco et al. 2021). After measuring usual alarm‐use rates (7% acute; 11% subacute), two units piloted alarm elimination and two piloted reduction to below 3%. This study informed a later RCT investigating alarm elimination and reduction (Pu et al. 2025). Prior to the disinvestment intervention, baseline rates across the 18 participating wards ranged from 3.39% and 24.45% of patients using alarms. The RCT showed high alarm use did not produce significantly lower fall rates than reduced or eliminated use. U.S. hospitals are also beginning to investigate alarm disinvestment. In an ongoing trial, a theory‐based, stakeholder‐driven disinvestment implementation package was developed using staff feedback only—patients and families were not included in the study sample (Turner et al. 2025).

### 4.4 Why Alarms Are Used

All studies across hospital and RAC settings suggested that the primary motivator to use alarms was to avoid patient harm related to falls, particularly in the hospital setting. Some studies raised other harms such as financial harm to the organisation (Fehlberg et al. 2018; Grealish et al. 2019; Mitchell et al. 2018; Shever et al. 2011), and harm to the organisation and staff in terms of legal liability and meeting regulatory requirements (Fehlberg et al. 2020; Lopez et al. 2010; Matsuo et al. 2008; Stephen, Pu, Weller‐Newton, and Haines 2026). In this regard, the effect of policy change with financial implications from falls for hospitals was quite pronounced. The Centers for Medicare and Medicaid Services (CMS) policy change in 2008 classified inpatient falls as ‘never events’ and eliminated reimbursement for fall‐related hospital inpatient costs (Centers for Medicare and Medicaid Services 2008). This fuelled organisational motivations to prioritise falls prevention. Eight (30%) of the U.S. hospital studies explicitly referenced the CMS policy change as context to their research. In the decade preceding 2008, no U.S. hospital‐based studies were published. By contrast, 13 such studies were published in the following decade. Local level hospital policies also triggered alarm research. Policies specifying that high fall risk patients must remain within an arm's length of a staff member when toileting motivated researchers to trial a toilet alarm (Jones et al. 2021).

Policy changes related to the use of restrictive practices were also influential across hospitals and RAC. The U.S. Omnibus Budget Reconciliation Act of 1987 discouraged physical restraint use in RAC and led to the adoption of alarms as a perceived safer alternative (Crogan and Dupler 2014; Jagella et al. 1992). Vassallo et al. (2005) highlighted the ambiguity of restraint definitions, emphasising they did not differentiate between different fall prevention interventions. They found more patients and family members considered alarms a restraint (30%) compared to staff (21%). A policy shift with restraints occurred in 2017 whereby CMS reclassified alarms as a form of passive restraint due to feelings of restricted freedom to move and advised restricting their use within RAC (Hartmann et al. 2021; Okumoto et al. 2020). Within the RAC setting, Europe was quicker to adopt policies recognising alarms as a form of restraint, but this did not equate to alarm disinvestment. In Zwijsen's study (Zwijsen et al. 2012), only 18% of staff viewed alarms as a restraint and used alarms to supplement physical restraints, rather than replace them. Staff felt less secure if only alarms were used compared to restraints. Nurses and families sometimes felt there was no option but to use restraint and were perplexed doctors believed restraints (inclusive of alarms) should not be used in these situations.

Motivations to research alarms differed between hospital and RAC settings. Some hospital‐based studies cited limited evidence for alternative fall‐prevention strategies as justification to investigate mobilisation alarms (Dollard et al. 2022; Ferrari et al. 2012). Others claimed there was sufficient evidence for how to prevent hospital falls and acknowledged the uncertain evidence supporting alarm effectiveness. Despite this, they argued that alarm use should continue to be promoted given clinicians' strong reliance on alarms, ongoing fall rates, and alarms' relatively low cost and straightforward use (Anderson and Breda 2025; Johnston and Magnan 2019). In RAC, discussion of alarm effectiveness was noticeably absent except in disinvestment studies; rather, research was centred more on the issue of whether alarms were a form of restrictive practice (Zwijsen et al. 2011, 2012).

### 4.5 Attitudes Among Staff, Patients, Residents and Family Members Towards Alarms

Twenty‐nine (50%) studies addressed only staff perspectives. Six (11%) studies, all conducted in hospital, exclusively addressed patient or family perspectives. Residents' perspectives were largely absent from RAC research. Thirteen studies incorporated some patient, family or resident views alongside staff perspectives, though some were limited in analytical depth and perspectives presented only briefly (Tideiksaar et al. 1993; Kelly et al. 2002; Matsuo et al. 2008; Widder 1985; Wolf et al. 2013).

Five themes were constructed across hospital and RAC studies pertaining to attitudes towards alarms: effectiveness, workload impact, liability concerns, device knowledge and ethical tensions (Figure 2). These themes were interconnected. Grounded in the ABC model of attitudes (comprising affect, behaviour and cognition) (Haddock and Maio 2008), device knowledge represents the cognitive domain, forming the foundational understanding and beliefs that directly inform perceptions of alarm effectiveness and whether alarms added to or reduced workload. Liability concerns added to workload and contributed to ethical tensions as staff weighed the risks and benefits of alarm use. Refer to Table S4 for studies and quotes depicting themes.

**FIGURE 2:**
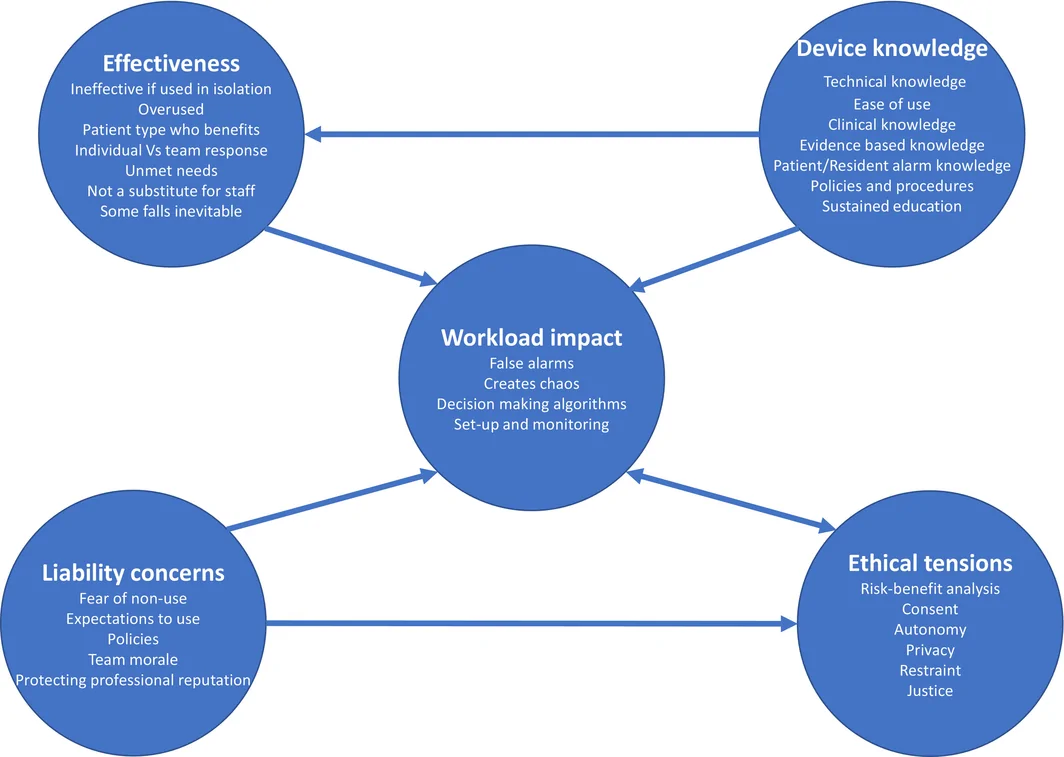
Themes shaping attitudes towards alarms. [Colour figure can be viewed at wileyonlinelibrary.com]

#### 4.5.1 Alarm Effectiveness

Staff and patients expressed varying perspectives on alarm effectiveness (Hubbartt et al. 2011; Stephen and Campbell 2023), while rehabilitation experts classified them as ‘not beneficial but not harmful’. (Sarkies et al. 2019, 6) Despite a lack of evidence supporting their effectiveness, they remained a favoured fall prevention strategy among some staff, with claims they were ‘vital in keeping them (residents) safe’. (Hall et al. 2017, 65) Analysis of 67 nurses' post‐fall reflections revealed alarms were overwhelmingly the most frequently suggested prevention strategy (Hoke and Zekany 2020) and small‐scale observational studies demonstrated staff believed the alarms tested were effective (Wong Shee et al. 2014; Diduszyn et al. 2008; Matsuo et al. 2008; Subermaniam et al. 2016; Wolf et al. 2013; Yesmin et al. 2022). Other staff disputed alarm effectiveness, reasoning that alarms did not resolve patients' underlying needs (Hubbartt et al. 2011). Staff and patients across hospital and RAC doubted alarms alone prevented falls and emphasised the need for additional interventions (Barker et al. 2017; Stephen and Campbell 2023; Zwijsen et al. 2012).

Perceived alarm effectiveness depended on alert response time (Dollard et al. 2022; Considine et al. 2023; Stephen, Pu, Weller‐Newton, and Haines 2026). Alarms were considered helpful if all staff were expected to respond, regardless of patient allocation (Fehlberg et al. 2020; Porter et al. 2018). Examples were provided in hospital (Clodfelter 2025) and RAC (Vandenberg et al. 2017) where the customisable nature of the alarm positively impacted effectiveness by improving staff response. However, if alarms were overused, staff were less committed to respond (Barker et al. 2017; Fehlberg et al. 2020). Knowing residents well assisted prioritising alarm responses (Zwijsen et al. 2012), but this concept was less acknowledged in hospital. Staff perceived alarms were needed because of insufficient staffing, but also thought they did not work because too few staff were available to respond quickly enough to prevent falls (Considine et al. 2023; Porter et al. 2018; Stephen, Pu, Weller‐Newton, and Haines 2026).

Perceived alarm effectiveness was also related to patient type (Stephen and Campbell 2023; Timmons et al. 2019). Staff wanted to override the random alarm allocation in a RCT believing they could better identify fall risk and allocate alarms more appropriately (Timmons et al. 2019). Staff thought alarms were effective for patients/residents who moved quickly (Hubbartt et al. 2011) or knew to stop mobilising upon hearing the alert (Kelly et al. 2002; Widder 1985). However, patients reported alarms rarely reminded them to call for assistance before mobilising (Radecki et al. 2018). Alarms were believed to contribute to falls in some patients/residents by increasing agitation and confusion (Considine et al. 2023; Stephen, Pu, Weller‐Newton, and Haines 2026; Zwijsen et al. 2012). Occasionally, alarms could replace constant patient observers, but generally agitated patients were felt to require more observation than what alarms could facilitate (Hubbartt et al. 2011). Stroke survivors expressed constant observation was the most effective way to prevent patients like themselves falling, but if observation was not feasible then an alarm was perceived as the next effective option (Gray 2013).

Across hospital and RAC, some staff and patients expressed nihilistic attitudes towards falls—some people would fall regardless of whether alarms were used or not (Considine et al. 2023; Hoke and Zekany 2020; Stephen and Campbell 2023). Nevertheless, alarms were felt effective in alerting staff when falls occurred to prevent complications from long lies (Radecki et al. 2018; Stephen, Pu, Weller‐Newton, and Haines 2026). In contrast, families often believed alarms almost guaranteed falls would not occur (Hall et al. 2017; Stephen, Pu, Weller‐Newton, and Haines 2026; Zwijsen et al. 2012).

#### 4.5.2 Workload Impact

Overall, alarms were perceived to negatively impact workload due to alarm overuse. Hospital managers' attitudes towards falls determined whether alarms helped or hindered workflow. Hospitals are fast‐paced, stressful and busy—intensified by rigid adherence to organisational goals of zero falls. This resulted in an overreliance on alarms, contributing to a chaotic atmosphere (King et al. 2018). Some hospitals instituted approaches such as ‘guilty unless proven innocent’ whereby all patients had alarms activated unless they were ‘proven innocent’ and assessed as safe to mobilise independently (Fisher et al. 2014, 17). RAC showed similar impacts. Where the culture prioritised individualised care and managers had a more relaxed attitude towards falling, customisable alarm devices enabled staff to care for residents in a calm manner including effective alert responses. However, when the culture was one of collective vigilance, fear of falls and use of non‐directional alarm systems, alarms increased staff workload as ‘everybody's supposed to be listening’ leading to uncoordinated staff responses (Vandenberg et al. 2017, 278). Nevertheless, customising alerts to designated staff was not universally preferred. Some staff felt this overburdened a small number of staff and favoured alerts configured throughout the building (Hall et al. 2017).

Staff reported numerous workflow concerns regarding daily alarm use: alerts were noisy and disruptive (Considine et al. 2023), additional time was required to set up and monitor alarms (Hubbartt et al. 2011), patients intentionally used alarms to ensure a rapid response (Radecki et al. 2018), and alarm fatigue due to excessive false alerts (Barker et al. 2017). Despite alarms' negative consequences on nurses' workloads, there did not appear to be any overt desire from frontline staff to change current alarm practice in hospital, except one example where the responsibility of alarm pagers was delegated to healthcare assistants so nurses were not disturbed (Timmons et al. 2019). In RAC, staff occasionally turned alarms off, did not replace batteries or claimed pagers were broken to avoid responsibility for them (Hall et al. 2017; Iio et al. 2016). In contrast, those committed to using alarms devoted extra time to perform additional rounding with the sole purpose to ensure alarms were working properly and positioned correctly (Zwijsen et al. 2012). Overall, nurses across hospital and RAC recognised the intent of alarms was to improve patient surveillance to prevent falls, thus they seemed prepared to shoulder the extra workload associated with alarms if it meant an opportunity to prevent a fall was not missed.

In contrast to these qualitatively reported daily workflow disruptions, quantitative evaluations measuring staff attitudes revealed more positive workload impacts. Initial staff concerns about the additional workload alarms might impose dissipated over time (Timmons et al. 2019; Wolf et al. 2013). In pre‐post studies that tested integrated bed alarms, most staff reported they reduced workload and freed time for other work (Subermaniam et al. 2016; Wen et al. 2022). Whilst staff rated the bed alarm the most time‐consuming and cumbersome, it was not the most annoying, leading the authors to conclude staff wanted to be notified by this type of alarm (Wen et al. 2022). Most staff trialling a toilet alarm in another pre‐post study agreed it gave patients more privacy (82%) and was safer (88%), but its positive impact on staff productivity (52%) and daily workflow (60%) was less convincing (Jones et al. 2021).

#### 4.5.3 Liability Concerns

High alarm use in hospitals was often driven by liability fears. Staff feared their professional reputation was at risk, believing their organisations might not defend them if their decisions not to use alarms were challenged (Stephen, Pu, Weller‐Newton, and Haines 2026). Staff believed managers and families expected alarms to be used and asked whether they were used post falls. Staff also interpreted hospital policies as mandating their use (Stephen, Pu, Weller‐Newton, and Haines 2026; Turner et al. 2025). Likewise, patients felt they needed to comply with alarms, perceiving them as part of hospital policy (Stephen and Campbell 2023). Some staff doubted alarm effectiveness, but fear of punishment led to compliancy‐driven alarm use rather than individual‐based patient assessment (Fehlberg et al. 2020; Stephen, Pu, Weller‐Newton, and Haines 2026). Overwhelming fears of liability created tension around alarm decisions and who was and was not responding to alarm alerts, which contributed to low team morale (King et al. 2018; Stephen, Pu, Weller‐Newton, and Haines 2026).

Perceived pressure to use alarms and liability fears were less apparent in RAC. In fact, the opposite existed. RAC policies motivated staff not to use alarms by presenting evidence of alarms' inappropriateness and focused on negotiating risk and benefits of alarms. Consequently, staff did not need to fear liability when patients fell when alarms were not used (Vandenberg et al. 2017; Gordon et al. 2015; Hartmann et al. 2021).

#### 4.5.4 Applied Device Knowledge

Alarm use was influenced by staff's technical knowledge of operating the device, clinical knowledge of knowing when and why to use (or not use) it, and finally, knowledge of research evidence regarding alarm effectiveness. Staff knowledge of alarm evidence scored the lowest when compared to two other health interventions (Sarkies et al. 2019). A lack of evidence‐based knowledge among hospital leaders and staff created a barrier to alarm disinvestment (Turner et al. 2025). Staff appeared to assign greater importance to organisational fall prevention policy adherence compared to published evidence. Several studies (Hoke and Zekany 2020; King et al. 2018; Porter et al. 2018) highlighted the role of fall prevention policies in educating staff in appropriate alarm use.

Shorter hospital admissions limited the time available to evaluate patients' potential to safely mobilise unassisted (Hubbartt et al. 2011). Less experienced staff perceived prescriptive alarm policies valuable because they could quickly identify when a patient required an alarm. This created a tension whereby more experienced nurses believed they should have autonomy to choose when to adopt alarms or not, rather than stringent policy directives (Considine et al. 2023; Fehlberg et al. 2020; Stephen, Pu, Weller‐Newton, and Haines 2026). Some hospital staff were unaware they had a fall prevention policy; instead, they perceived management guidance as three simplistic steps: identify patients at risk of falls, place alarm on and run to alarms (King et al. 2018). Lack of a fall prevention policy, or knowledge of one, led to alarm overuse and contributed to low morale as falls continued and staff lacked knowledge of alternative approaches.

Some alarms were reported to be non‐intuitive to use (Lopez et al. 2010), implying a need for education. The importance of including casual or new staff in alarm education was highlighted (Wong Shee et al. 2014). Staff attributed user errors, such as not docking devices to charge, alarm non‐use, incorrect sensitivity settings, and lack of staff knowledge of expected response, as potential contributors to falls and excessive alarm fatigue (Clodfelter 2025; Hoke and Zekany 2020; Matsuo et al. 2008). Expectations appeared to be ‘everyone’ responded to alarm alerts; however, it was uncertain whether non‐nurses within the inter‐professional team knew to respond (Porter et al. 2018; Stephen, Pu, Weller‐Newton, and Haines 2026). It was recommended alarm policies included: indicators for use/non‐use, appropriate alarm parameter settings and expected staff response to alerts. Alarm educational interventions were developed in response to evidence that staff were consistently using alarms improperly (Anderson and Breda 2025; Skolka et al. 2023). These small pre‐post studies were not designed to investigate alarm effectiveness; however, they claimed a positive association between improved staff knowledge of alarm policies and reduced patient falls.

Not all studies reported staff difficulty with alarm use, suggesting that alarm education was sometimes adequate. One of the first RCTs to investigate alarm effectiveness reported nurses found the system user friendly, easy to install and operate (Tideiksaar et al. 1993). More recently, pre‐post studies have specifically evaluated the usability of three‐stage integrated bed alarms. One study found 96% of staff rated them ‘easy to operate’. (Subermaniam et al. 2016) Another study was less convincing—only 44% agreed it was ‘easy to use’, (Wen et al. 2022) whilst another study indicated staff were ‘satisfied overall’ with the device (Balaguera et al. 2017). Other staff encountered difficulty adjusting the many bed parameters, highlighting the need for proper education of all integrated ‘smart’ bed components (Yesmin et al. 2022).

Patients' alarm knowledge appeared limited in the few studies that explored this. Widder (Widder 1985, 9) highlighted ‘nurses' careful explanation of the function and purpose of the device before use was essential in gaining patient cooperation’. However, there was limited literature explaining the best way to do this, particularly for those with cognitive impairment, who are often the ones receiving alarms. Some patients appeared unaware of the alarm and nurses rarely acknowledged to patients that it was the alarm alert that instigated them attending the bedside (Timmons et al. 2019). Alarm education to patients with cognitive impairment was often difficult (Hall et al. 2017; Stephen, Pu, Weller‐Newton, and Haines 2026). Sometimes it was assumed alarm explanations were not required due to their limited ability to understand. However, patients with cognitive impairment demonstrated an awareness when alarms were in use and more explicit education may have avoided patients misinterpreting the alarm's purpose (Dollard et al. 2022; Stephen and Campbell 2023).

Device knowledge was not prominent in the RAC research. This may be due to most RAC studies focussing on alarm disinvestment and, apart from one study (Kelly et al. 2002), none researched implementation of a new alarm. The disinvestment research outlined the structured education programs considered essential to disinvest from alarms. Formalised education sessions, role playing, newsletters, policies, and the use of compelling evidence questioning alarms' effectiveness were used for staff and families to increase knowledge and change expectations that alarms were essential (Bressler et al. 2011; Crogan and Dupler 2014; Hartmann et al. 2021).

#### 4.5.5 Ethical Tensions

A key aspect of ethical practice is ensuring the intervention, such as an alarm, provides more benefit than harm. Ethical tensions existed in the hospital‐based studies even if they were not overtly acknowledged by participants or researchers. Educational interventions regarding alarms centred almost exclusively on technical aspects and role responsibilities, omitting guidance on how to negotiate the ethical dilemmas (Anderson and Breda 2025; Skolka et al. 2023). Consequently, staff qualitatively reported tensions with the safety‐risk conflict they must negotiate—the potential benefit of preventing a fall, opposed to the risk of increasing agitation, distress or immobility and subsequently escalating fall or pressure injury risk (Considine et al. 2023; Fehlberg et al. 2020; Stephen, Pu, Weller‐Newton, and Haines 2026). A disconnect emerged between survey evaluations and patient experiential accounts. Quantitative survey measures predominantly rated alarms as comfortable, unrestrictive and non‐intrusive (Dollard et al. 2022; Ferrari et al. 2012; Jones et al. 2021; Shinmoto Torres et al. 2017). In contrast, qualitative accounts revealed that negative experiences (feeling offended, restricted or subjected to unnecessary surveillance) far outweighed positive feelings of reassurance (King et al. 2021; Radecki et al. 2018; Stephen and Campbell 2023; Timmons et al. 2019).

Practices differed in RAC regarding who negotiated, and whether they negotiated, the benefits and harms of alarms. This was demonstrated in multiple‐case studies with conflicting facility cultures and willingness to engage in ethical alarm debates (Vandenberg et al. 2017; Hall et al. 2017). RAC facilities that lacked ethical discussions experienced falls as a ‘huge’ problem and overused alarms with little individualisation to residents' preferences and routines. In contrast, RAC facilities that encouraged debate and had high involvement of staff, family and residents in alarms decisions described the issue of falls as ‘less important than taking people's freedom away’. (Vandenberg et al. 2017, 279).

Another aspect of ethical practice is justice—a fair distribution of benefits and burdens. Nurses raised concerns that patients using alarms took care away from others not using alarms (Considine et al. 2023). Examples included where patients deliberately set off their alarm to ensure expedited assistance (Radecki et al. 2018; Timmons et al. 2019). Nurses tasked with multiple alarm responsibility frequently experienced the unjust dilemma of choosing who to respond to during simultaneous alarms or leaving a high‐risk patient unattended (Porter et al. 2018; Stephen, Pu, Weller‐Newton, and Haines 2026; Timmons et al. 2019).

The participant perspectives researched also reflected an injustice. Alarms are commonly used by patients experiencing cognitive impairment, but the majority of studies focused on staff perspectives. Studies that sought patients' perspectives either excluded those with cognitive impairment (Radecki et al. 2018), had inclusion criteria that made it difficult for those with cognitive impairment to participate such as remembering previous hospital admissions in the last 12 months (King et al. 2021), only had a small percentage of patients with cognitive impairment (Dollard et al. 2022) or lacked detail of patient participant characteristics (Timmons et al. 2019). Only one study included a sample whereby all participants had some degree of cognitive impairment (Stephen and Campbell 2023). Importantly, some authors raised lack of inclusion of patients with cognitive impairment as a study limitation (Ferrari et al. 2012; Hall et al. 2017).

Patient autonomy was raised in regard to patient preference and consent. The importance, but difficulty, of including patients with cognitive impairment in shared decision making was highlighted (Dollard et al. 2022; Stephen and Campbell 2023; Stephen, Pu, Weller‐Newton, and Haines 2026). Patient choice not to have an alarm was sometimes respected (Hoke and Zekany 2020; Stephen and Campbell 2023; Timmons et al. 2019), however, there were also instances where patient preference was overridden (Diduszyn et al. 2008; Stephen and Campbell 2023; Stephen, Pu, Weller‐Newton, and Haines 2026). Widder's (Widder 1985) statement about a manufacturer changing the thigh sensor attachment to hinder removal perhaps reflects the historical context in which patient preference was less respected. A small pilot RAC study of a thigh‐mounted alarm observed six residents (13%) requested alarm removal due to the device limiting their ability to mobilise unsupervised (Kelly et al. 2002). Whilst wearable alarms are designed to be worn unnoticed to promote dignity, acceptance is still closely related to autonomy. The authors recommended this alarm unsuitable for people who valued autonomous mobility.

The ethics of restraint was addressed in hospital (Okumoto et al. 2020) and RAC (Vassallo et al. 2005). Key considerations were highlighted on RAC restraint reduction inclusive of alarms (Zwijsen et al. 2011). First, doctors were authorised to make restraint decisions, but nurses were responsible for implementing those decisions in practice. Nurses held conflicting views of what constituted a restraint. Some nurses considered a device a restraint only if it caused residents discomfort, restricted freedom, or invaded privacy. Second, restraint use should be regularly evaluated and satisfactory alternatives discussed in a way that avoided accusatory implications. Lastly, perspectives of residents should be considered, acknowledging the difficulty, but not impossibility, for those with dementia.

Finally, respecting privacy was a dominant theme in RAC, but less prevalent in hospital. There were conflicting views whether alarms invaded privacy or benefited it. RAC staff argued they could provide care ‘from a distance’ and alarms directed their attention to where it was most needed at the right time, promoting good quality care (Hall et al. 2017; Zwijsen et al. 2012, 218). Within the hospital setting, staff reported patient frustration at alarms going off, leading to staff ‘helicoptering and managing them’. (Stephen, Pu, Weller‐Newton, and Haines 2026, 10).

## 5 Discussion

This scoping review sought to identify staff, patient, resident and family attitudes towards the use and benefits of alarms in falls prevention across hospital and RAC. To our knowledge, this review is the first to investigate the scope of evidence specifically encompassing perspectives of alarms across both settings. Research in this area is heavily skewed towards hospitals and staff perspectives, with limited research specifically dedicated to RAC as well as patient, resident, family and non‐nursing perspectives. Nurses' attitudes towards alarms were well represented in the literature and probably do not require further research; however, there is still a need for nurses' perspectives on alarm disinvestment or emerging falls prevention technology where usability, workflow integration and ethical implications require further investigation. The majority of research evaluated traditional pressure‐sensitive alarms; however, as technology advanced, integrated bed alarms gained traction yet did not replace traditional alarms. Overall, this scoping review highlighted a paradox regarding recent research which focuses both on optimising alarm use and on disinvesting from them, highlighting the ongoing ambiguity of their role in falls prevention in hospital and RAC.

Many factors influenced whether people perceived alarms effective and helpful, or ineffective and burdensome. Knowledge of device use, perceived expectations to use and organisational policies shaped how often or how effectively alarms were implemented. Hospital alarm use tended to be driven by liability fears. Nurses describe feelings of anguish, guilt, failure and reputation risk following a patient fall; hence, alarms are viewed as a rapid, accessible safeguard against these lasting emotional impacts (Bok et al. 2016; de Quadros et al. 2022). A stark omission in the literature is the perspective of allied health professionals and doctors towards mobilisation alarms given falls prevention is explicitly framed as ‘everyone's responsibility’ (Australian Commission on Safety and Quality in Health Care 2025). Multidisciplinary ownership is essential, particularly because sharing responsibility across disciplines reduces perceived individual liability (Singh et al. 2022), a key driver of alarm use found in this review.

Feelings of guilt when a patient falls without an alarm may be misguided given that meta‐analyses show that high rates of alarm use do not reduce fall rates (Haines et al. 2026). Although an RCT showed some uncertainty remains about eliminating alarms entirely (Pu et al. 2025), current high rates of use diverge from this high‐level evidence. Based on such evidence and this scoping review findings that staff feel organisation tools implicitly mandate alarm use, hospital managers should consider removing alarms as a suggested strategy in their organisation falls guidelines and integrated risk assessment and management tools (Stephen, Pu, Weller‐Newton, and Haines 2026). Hospital falls clinical practice guidelines have not recommended alarms since 2011 (McKercher et al. 2024), yet local hospital tools and risk assessments continue to feature them. These tools act as cognitive heuristics, enabling staff to make rapid decisions under time pressure or cognitive fatigue (Bowes et al. 2020). However, this creates a ‘doubled‐edged sword’, standardising care to protect staff against organisational blame, but their rigidity can perpetuate alarm overuse misaligned with evidence‐based practice when patient complexity warrants deviating from suggested recommendations (Khaled Al Abdulqader et al. 2025).

Hospital research was predominantly conducted in the U.S. No research was completed in low‐income countries. Expanding mobilisation alarm research internationally is important given healthcare systems, liability concerns, and cultural attitudes towards alarms are likely to vary across countries. For example, in many Asian countries, ‘hands‐on’ family involvement in care processes is a cultural norm and responsibility, Western countries lack this expectation and consequently may rely on technology such as mobilisation alarms to fill the gap (Park et al. 2022). A counterpart scoping review on video monitoring for falls prevention demonstrated its growing popularity, similarly, concentrated in U.S. hospitals rather than more globally or across RAC settings (Stephen, Pu, Weller‐Newton, Manohar, and Haines 2026). Unlike traditional mobilisation alarms designed solely to prevent falls, video monitoring is driven by multiple organisational motives, including workforce optimisation via reducing constant observers, cost efficiency or user experience, which complicate how its overall effectiveness is judged. Ultimately, hospital staff still preferred dedicated one‐to‐one in‐person patient observers over video monitoring for falls prevention.

Another type of mobilisation alarm is ‘wearables’. This scoping review search retrieved some experimental studies that evaluated wearable accelerometer alarms with the purpose of falls prevention and found them acceptable, yet none expanded use into usual clinical care. Reasons for this lack of translation were not documented, but it is hypothesised that this area will continue to grow given technological advancement in wearables.

There was limited research exploring drivers of alarm use in RAC beyond them being a more acceptable alternative to physical restraint. RAC research pivoted to alarm disinvestment in response to consideration that alarms themselves could be viewed as a form of restraint (Bressler et al. 2011; Crogan and Dupler 2014; Hartmann et al. 2021). In response to the Royal Commission into Aged Care Quality and Safety, Australia introduced a rights‐based approach into aged care legislation (Australian Government Department of Health, Disability and Ageing 2025). However, operationalising rights‐based care remains challenging when residents lack the capacity or agency to assert their preferences. Research indicates residents are more willing to express concerns to an independent person (i.e., the researcher) than with staff (Kingston and Luke 2026). Without independent advocacy, there remains a risk that alarms are introduced or maintained against resident wishes.

The lack of involvement of adults with cognitive impairment in research about alarms for falls prevention is particularly salient given their vulnerability to falls (Zhang et al. 2019) and the primary recipients of alarms. Tailored patient education is one of the few interventions shown to have robust evidence in preventing falls (Montero‐Odasso et al. 2022). However, it provides limited benefit for those with cognitive impairment (Haines et al. 2011) leaving a major gap in evidence regarding falls prevention for cognitively impaired adults and that specifically incorporates their perspectives. Although including this vulnerable population's perspectives has its methodological challenges, it is by no means impossible. Utilising process consent methods (Dewing 2007), having researchers skilled in interviewing older adults with cognitive impairment (Samsi and Manthorpe, n.d.), and incorporating observation methods to systematically observe nonverbal responses to alarms for those unable to verbalise their experiences is recommended. Ultimately, understanding end‐user perspectives can assist staff ethically balance the benefits and risks of alarms.

Only one quarter of the included studies were qualitative. Further qualitative research capturing the experiences of alarm disinvestment would be valuable given the complex phenomena of institutional fall prevention and may help guide healthcare leaders who acknowledge the limited evidence supporting alarms but lack clear roadmaps for disinvesting from high‐use. Evidence is also lacking on the perspectives of patients, residents and their families who are not using alarms but are admitted to high‐alarm units to understand the broader environmental impact of alarm fatigue. Personal characteristics of the research team in the included studies were rarely reported, which impacts the transparency and credibility of findings. Future research on perspectives of alarms (including disinvestment) should incorporate statements of researcher reflexivity.

Given the lack of RAC studies, specifically recent alarm prevalence studies, it remains unknown whether early RAC alarm disinvestment research was sustained or translated across other RAC facilities. Such knowledge would be valuable to hospital staff who appear to be at the beginning of this disinvestment journey (Pu et al. 2025). The difference in time periods between the two settings embracing alarm disinvestment may be reflective of the well‐known evidence‐to‐practice translation gap, which progresses more slowly in mistrustful communities or under‐resourced health systems, noting that the early RAC disinvestment studies were conducted prior to the rise of implementation science expertise (Thomson et al. 2025).

### 5.1 Limitations

Several limitations of this review should be noted. Relevant studies may have been omitted despite our comprehensive search strategy, particularly given how rapidly research on disinvestment and novel technologies is evolving. Including quality improvement projects, alongside more rigorously conducted research, allowed a fuller understanding of the real‐world implementation of alarms. However, our data charting highlighted several recurring methodological limitations across the literature such as small sample sizes, limited qualitative depth and exclusion of perspectives from adults with cognitive impairment (although acknowledging a formal critical appraisal was not conducted). Future systematic reviews focussing strictly on rigorously conducted primary studies (excluding quality improvement projects) may help inform more tangible evidenced‐based clinical recommendations.

## 6 Conclusion

This scoping review presents an overview of the literature of why and how mobilisation alarms are used and attitudes towards their effectiveness in falls prevention within hospital and RAC settings. Uncertain evidence regarding the effectiveness of traditional pressure‐sensitive alarms, negative attitudes towards them and the emergence of alternative mobilisation alarms warrants future research efforts to concentrate on whether and how hospitals and RAC facilities have successfully disinvested in traditional pressure‐sensitive alarms. Importantly, this should include patient/resident, family and staff perspectives of any new ways of working or devices used when alarms are not being relied on, to understand their acceptability and sustainability for all stakeholders.

## Author Contributions

All authors meet the authorship criteria and approve the content of the manuscript. Conceptualization and methodology: K.S., D.P., J.W.‐N., R.M. and T.P.H. Data collection: D.P., R.M. and K.S. Data curation: K.S. Formal analysis: K.S., D.P., J.W.‐N. and T.P.H. Writing original draft: K.S. Visualisation and Review/editing of manuscript: K.S., D.P., J.W.‐N., R.M. and T.P.H. Supervision: D.P., J.W.‐N. and T.P.H.

## Funding

This work was supported by PhD scholarship funding from the Centre of Research Excellence in the Prevention of Fall‐related Injuries (APP1198371) for KS.

## Conflicts of Interest

The authors declare no conflicts of interest.

## Supporting information


**Table S1:** Eligibility criteria.

**Table S2:** (a) Search strategy. (b) Example of Medline search results.

**Table S3:** Study summary table.

**Table S4:** (a) Number of studies contributing to attitude themes. (b) Key quotes depicting themes.

**Data S1:** Preferred Reporting Items for Systematic reviews and Meta‐Analyses extension for Scoping Reviews (PRISMA‐ScR) Checklist.

## Data Availability

The data that supports the findings of this study are available in the Supporting Information of this article.
