# Supplementary material for: How Staff, Patients, Residents and Families Perceive Mobilisation Alarms for Preventing Falls in Hospital and Aged Care: A Scoping Review
Source: J Clin Nurs. 2026 Aug 5;35(12):5204–22. doi: 10.1111/jocn.70486 (PMC13744243; doi:10.1111/jocn.70486)
Supplement: Supplementary file 2 [file JOCN-35-5204-s002.docx]

**Supplementary table 1. Eligibility criteria**

|  | **Include** | **Exclude** |
| --- | --- | --- |
| **Population** | Hospital staff (all disciplines)  Patients  RACF staff (all disciplines)  Residents  Healthcare students  Families of patients/residents | People living in community (not RACF)  Children |
| **Interest**  How and why alarms are used and attitudes towards their use and effectiveness | Studies describing how alarms are used.  Any alarm type with the purpose of falls prevention: pressure sensitive, pull string, body worn, laser, video  Broader studies investigating falls prevention where alarms are not the primary focus, provided alarm practice/attitudes are separately reported | Alarms without the purpose of falls prevention such as falls detection (e.g. senses the person fallen on the floor), bed wetting alarms, vital sign alarms)  - Alarm effectiveness with no reporting of why/how alarms are used or attitudes towards their effectiveness |
| **Context** | Hospital – emergency, acute, subacute  transition care  RAC | Laboratory experimental studies  Private homes  Retirement Living |
| **Study design** | Qualitative  Quantitative (e.g. survey on acceptability of alarms)  Mixed method | Systematic reviews  Non-research opinion papers from experts providing “clinical wisdom” |
