# Supplementary material for: How Staff, Patients, Residents and Families Perceive Mobilisation Alarms for Preventing Falls in Hospital and Aged Care: A Scoping Review
Source: J Clin Nurs. 2026 Aug 5;35(12):5204–22. doi: 10.1111/jocn.70486 (PMC13744243; doi:10.1111/jocn.70486)
Supplement: Supplementary file 3 [file JOCN-35-5204-s003.docx]

**Supplementary table 4a. Number of studies contributing to attitude themes**

| **Theme** | **N studies** | **% of attitude studies**  **(Total 48)** | **Relevant studies contributing to theme** |
| --- | --- | --- | --- |
| **Effectiveness** | 30 | 63% | Barker (2017); Bressler (2011); Considine (2023); Clodfelter (2025); Crogan (2013); Diduszyn (2008); Dollard (2022); Fehlberg (2020); Gordon (2015); Gray (2013); Hall (2017); Hartmann (2021); Hoke, (2020); Hubbartt (2011); Ilo (2016); Kelly (2002); Matsuo (2008); Porter (2018); Radecki (2018); Sarkies (2019); Stephen (2023); Stephen (2025); Subermaniam (2017); Tzeng (2015); Timmons (2019); Vandenberg (2017); Widder (1985); Wong-Shee (2014); Yesmin (2022); Zwijsen (2012) |
| **Workload impact** | 18 | 38% | Barker (2017); Considine (2023); Diduszyn (2008); Fehlberg (2020); Fisher (2014); Hall (2017); Hubbartt (2011); Ilo (2016); King (2018); Lopez (2010); Matsuo (2008); Radecki (2018); Stephen (2025); Subermaniam (2017); Timmons (2019); Wen (2022); Wolf (2013); Zwijsen (2012) |
| **Liability concerns** | 13 | 23% | Bressler (2011); Considine (2023); Crogan (2013); Fehlberg (2020); Gordon (2015); Hall (2017); Hartmann (2021); Hubbartt (2011); King (2018); Stephen (2023); Stephen (2025); Turner (2025); Vandenberg (2017) |
| **Device knowledge** | 28 | 58% | Anderson (2025); Belaguera (2017); Bressler (2011); Clodfelter (2025); Considine (2023); Crogan (2013); Dollard (2022); Fehlberg (2020); Hall (2017); Hartmann (2021); Hoke, (2020); Hubbartt (2011); King (2018); Lopez (2010); Matsuo (2008); Porter (2018); Sarkies (2019); Skolka (2023); Stephen (2023); Stephen (2025); Subermaniam (2017); Tideiksaar (1993); Timmons (2019); Turner (2025); Wen (2022); Widder (1985); Wong-Shee (2014); Yesmin (2022) |
| **Ethical tensions** | 32 | 67% | Anderson (2025); Bressler (2011); Considine (2023); Crogan (2013); Diduszyn (2008); Fehlberg (2020); Ferrari (2012); Gordon (2015); Hall (2017); Hartmann (2021); Hoke, (2020); Ilo (2016); Jagella (1992); Jones (2021); Kelly (2002); King (2021); Okumoto (2020); Porter (2018); Radecki (2018); Skolka (2023); Stephen (2023); Stephen (2025); Tideiksaar (1993); Timmons (2019); Torres (2017); Vassallo (2005); Vandenberg (2017); Widder (1985); Wolf (2013); Wong-Shee (2014); Zwijsen (2011); Zwijsen (2012) |

**Supplementary table 4b. Key quotes depicting themes**

| **Theme** | **Quote** |
| --- | --- |
| **Effectiveness** | ‘We have more people on alarms than we have nurses that can manage them.’ Nurse, hospital (Considine, 2023)  ‘Even though they’ve got the [bed] sensors, people still fall… it’s just to alert you they’re getting out of bed.’ Care worker, RAC (Hall et al., 2017) |
| **Workload impact** | ‘Literally the CNS and nurse manager, they look at you and say, ‘I expect you to run into that room, I expect you to move faster’” "You’re just running down the hallways… We’re all scattered all over the place trying to run to these alarms.’ Nurse, hospital (King et al., 2018)  ‘It’s a big, big problem to have all these alarms going off… Sometimes, you know you go home a raving lunatic just from all the alarms you've heard all day.’ Hospital (Turner et al., 2025)  ‘If there are very unsettled people, then well, you can’t really use it during nights, because you’d be going to them every minute, and that would be unworkable.’ Nurse, RAC (Zwijsen et al., 2012)  ‘We have set up an out-of-bed sensor in the room of a dementia person. But, since he had wandered at night, we must have gone there again and again. We couldn’t get anything done without it. So, the switch of it was turned off.’ Care worker, RAC (Ilo et al., 2016)  ‘The system reacts too often, caregivers are tired.’ Resident, RAC (Ilo et al., 2016) |
| **Liability concerns** | ‘If it was to go to Coroners, or something like that, for those really, really bad cases, it makes me nervous about my registration and, how much the hospital will look after me … And if a bed alarm is something that could have been very easily implemented.’ Nurse, hospital (Stephen et al., 2025)  ‘I like it because this is a requirement from the hospital, I have to obey it.’ Patient, hospital (Stephen & Campbell, 2023) |
| **Device knowledge** | ‘Bed alarms were infrequently employed, non-intuitive to use, not audible at distances, and often deactivated and not reactivated.’ Field observations of nurses, hospital (Lopez et al., 2010)  ‘I don’t think there's a lot of time and space to think about the clinical reasoning providing an alarm.’ Allied health, hospital (Stephen et al., 2025)  ‘Our new grads [graduate nurses] think all the confused patient has to be on a bed chair alarm.’ ANUM, hospital (Considine et al., 2023)  ‘You have to stay vigilant, keep talking, keep educating. You can’t stop. It is a constant, every day, every day. And I haven’t stopped yet. We’re still talking about alarms.’ Nurse Manager, RAC (Hartmann et al., 2021) |
| **Ethical tensions** | ‘I found it really intolerable…I couldn’t even get out of bed. I mean, really, I had an actual alarm’ Patient, hospital (King et al., 2021)  ‘There can be six alarms going off. You can’t leave the person you’re with to go answer the other six alarms.’ Nurse, hospital (Porter et al., 2018)  ‘Until she loses the ability to make those decisions for herself then we’re not to intervene… people are entitled to make a bad choice.’ Manager, RAC (Hall et al., 2017) |
