# Supplementary material for: How Staff, Patients, Residents and Families Perceive Mobilisation Alarms for Preventing Falls in Hospital and Aged Care: A Scoping Review
Source: J Clin Nurs. 2026 Aug 5;35(12):5204–22. doi: 10.1111/jocn.70486 (PMC13744243; doi:10.1111/jocn.70486)
Supplement: Supplementary file 4 [file JOCN-35-5204-s004.docx]

**Supplementary table 3. Study summary table**

| **Author** | **Study aim** | **Key findings** |
| --- | --- | --- |
| Anderson & Breda 2025 | To address bed alarm knowledge gaps | - Increase in knowledge gained  - Alarm policy and educational sessions helpful |
| Balaguera et al. 2017 | To conduct a technology evaluation, including feasibility, usability, and user experience, of a medical sensor-based Intranet of things system in facilitating nursing response to bed exits | - Satisfied overall with user interface, system design, and phone notifications  - Specific alerts on patient statuses (ie, stirring, sitting up, leaving, out of bed) informative in understanding patient behaviors and comfort levels |
| Barker et al. 2017 | To explore the acceptability of the 6-PACK program from the perspective of nurses and senior staff prior to implementation of the program | - Alarms may not be effective if used in isolation  - Mixed beliefs about alarm benefit. Useful if used alongside other interventions  - Alarms annoying and therefore ignored, increased workload, often broken  - Authors recommended regular maintenance audits, education promoting use of alarms in combination with other interventions and guidelines about prioritising use to only 1-3 patients on a unit at a time |
| Bressler et al. 2011 | RACF to be “alarm free” | - Entire RACF became quieter / calmer for residents to live and staff to work  - Staff more attuned to residents’ needs once ceased to rely on alarms. When a patient attempted to exit a bed/chair unassisted, staff asked what the resident needed rather than telling them to sit back down  - Staff commented residents sleep better without alarms, less agitated and fewer behaviors |
| Brusco et al. 2021 | - Describe usual care alarm utilisation across six health services  - Compare alarm utilisation post policy introduction to reduce/eliminate alarms | **Average alarm utilisation across health services:** Acute: 7% ( n = 171/2338); Rehab: 11% ( n = 286/2623)  Reduced alarm condition utilisation rate: 1.8% (n = 11/609)  Eliminated alarm condition utilisation rate of 0.0% ( n = 0/521) |
| Clodfelter 2025 | To identify and prioritize sociotechnical factors and interventions that impact the end-to-end Bed Exit alarm workflow | - Rerouting calls to only assigned staff facilitated more intentional responses and improved system usefulness. Decreased phone and environmental noise. Wireless alerts reduced 75%. Fewer declines because fewer unnecessary alerts sent |
| Considine et al. 2023 | To explore nurses’ experiences of using bed and chair alarms | **Negative impacts of falls alarms:** noisy technology, imperfect technology  **Juggling the safety-risk conflict:** triggers behaviours of concern; creates fall risk; interrupts nursing care; other patients get neglected; patients still fall  **Negotiating falls alarm use:** nurse decision making; alarm overuse |
| Crogan & Dupler 2013 | To describe development and pilot testing of an alarm elimination program for nursing homes  - Will an alarm elimination training program lead to improved staff knowledge regarding use of alarms and reduction / elimination of alarms used? | Prevalence at baseline (Jan 2012) - post Ix (June 2012):  RACF A: 31/132 (23.4%) – 0; RACF B: 53/164 (32.3%) – 0; RACF C: 25/116 (21.5%) – 0; RACF D: 43/87 (49.4%) - 20 (22.9%)  Took 5 months for RACF D to be alarm free, no increase in falls/falls with injury |
| Diduszyn et al. 2008 | To describe our experience using the Posey Sitter II wireless nurse call fall monitor to reduce falls among elderly inpatients | - 91% thought alarm helped prevent falls  - Low number of false alarms when alarm used properly  - 80% said patient did not operate call bell to ask for help before alarm went off. However, 44% judged the patient had ability to operate it - might indicate nurses overestimated patient's ability to use call system. Or despite recommendation to use call bell, many people like to be independent and get up by themselves  - 10 min to program alarm system with patient's information - viewed as burdensome because already busy |
| Dollard et al. 2022 | To report on the acceptability, usability, and safety of AmbIGeM system from perspectives of patients before and after use (and informal carers for patients with cognitive impairment) | - Preventing Falls Using the AmbIGeM System Was Appropriate and Valuable (Acceptability)  - Experiencing Little Impact on Patient Experience (Usability)  - Enhanced Patient Safety  - Insufficient Participant Information and Understanding  - Participants considered AmbIGeM a good idea. Most but not all thought the singlet and sensor component acceptable and comfortable, with no privacy concerns. Participants felt reassured with extra monitoring, although sometimes misunderstood AmbI-GeM purpose of detecting falls |
| Fehlberg et al. 2018 | To examine whether the CMS no-pay policy influenced 4 fall prevention practices: bed alarms, sitters, room changes, and physical restraints | Medical order for alarm: Before CMS 31/1099; After CMS 40/760  - 89.8% increased odds of use of bed alarm (OR = 1.898, 95% CI = 1.174–3.069) after implementation of CMS policy change  - Odds of nurses’ use of a bed alarm increased 2.3 times (95% CI = 1.409–3.897) in models adjusted for covariates |
| Fehlberg et al. 2020 | To examine acute care RN’s fall prevention decision-making | **Fear of not adhering to procedures**: Implemented alarms to avoid “getting in trouble”  **Adequate staffing / patient workload:** Meeting needs of high fall risk patients; Increased demands for alarm monitoring  **Value of bed alarms:** Varying perspectives on alarms benefit; Team commitment to respond to alarms  **Trust with patients/families**: Using alarms until patients prove they call for assistance  **Risk versus benefit**: Risk of associated with alarms – reluctance to reposition themselves or specialty bed incompatibility |
| Ferrari et al. 2012 | - Are sensors acceptable to hospitalized older adults while performing common movements during 4 hours of wear?  - Do hospitalized older adults wearing sensors for 4 hrs. maintain skin integrity? | - Acceptance calculated from mean values of wrist, ankle, and chest sensor results. Overall acceptance after 4 hrs. of wear: 4.77 out of 5 |
| Fischer et al. 2014 | To review how a medical-surgical unit took a stand against falls by creating and sustaining a successful fall prevention program | Purchased chair alarms. Staff benefited from early detection of patient movement, and patients benefited from quick staff response. Both bed and chair alarms felt to be instrumental in decreasing unit’s fall rate  - Instituted a new rule, “guilty unless proven innocent.” Unlike other alarm systems that may sound a false alarms, staff found alarms sounded reliably only when a patient attempted to move from bed. Alarm sensitivity allowed for repositioning. Patients “proven innocent” were those assessed by nurse to safely ambulate independently and alarms were not engaged for them |
| Gordon et al. 2015 | To assess impact of stopping routine use of bed alarms | Staff indicated floors were quieter and less chaotic |
| Gray 2013 | To discuss stroke survivor/caregiver experiences with falls, review fall prevention strategies from our organization, and any suggestions to prevent stroke patients falling | Many verbalized as a result of their deficits e.g. impulsivity, memory changes or aphasia, they felt our current fall bundle would not prevent stroke patients from falling. They felt having someone in the room was best practice. If no one could be in the room, recommended using alarms |
| Grealish et al. 2019 | To describe nurses’ implementation of activities recommended in evidence-based guidelines for falls prevention and care of older people with cognitive impairment | - Turned on bed alarm: Yes: 8; Applicable: 14; Adherence: 57%  - Alarms ranked 3rd lowest (29th) in terms of adherence (Pain assessed 34% and Reorient using clock/calendar 21%) |
| Hall et al.  2017 | To explore facilitators and barriers to the uptake of monitoring technologies into routine practice in care homes  To explore:  - The ethical debate between ‘safety’ and ‘freedom’  - Perceptions of benefits using monitoring technologies balanced against potential challenges such as false alarms, and organisational processes | - Considerations of positive or negative impacts upon privacy or freedom of movement were slight compared to the need to keep residents physically safe  - Increased privacy from not needing continuous human observation. Directs staff attention to where it is most needed at the right time  - Staff recognised technologies do not guarantee safety. Less acknowledged among relatives. Relatives often favoured alarms to enhance safety even if it threatened privacy  - Managers cautious, citing ethical concerns about potential ‘Big Brother’ effect  - Staff feared blame for falls, despite no reports of this happening in RACF. Fears influenced by media portrayal of RACFs  **Ways in which technologies were implemented**  - Commonly perceived as simple to use, yet more formal training valued  - Documented monitoring decisions varied so inconsistent understanding which residents had alarms  - Attempted to respect resident choice. Challenges in ensuring residents fully aware of implications |
| Hartmann et al. 2021 | To study impressions of an alarm de-implementation and sustainment intervention | **Initiating De-implementation** - Compelling participants with evidence; Engaging local leadership; Site level education and training  **Changing Expectations** - Educating staff and family  **Using Contrasting Approaches** - Gradual alarm elimination or piloting; Abrupt elimination of alarms; No alarms on site  **Witnessing Positive Effects of De-implementation in falls** - Hard to correlate residents’ behavior with alarms because different initiatives started: being proactive, talking to residents’, sleep assessment, deep dives. Improved sleep; Reduction in distressing behaviors; Increased resident engagement; Reduction in falls  **Staying the Course** - Received some requests from staff to reintroduce alarms. Leaders emphasized importance of consistently sustaining alarm elimination, used guidelines to motivate staff and presented evidence of alarms’ inappropriateness  - Others described a more complete change in culture where alarms were seen as a thing of the past |
| Hill et al.  2022 | To decrease inpatient falls and injury by 30% within 6 months | - Alarm cords frequently plugged in incorrectly leading to alarm failure. Color-coding cords removed error risk |
| Hoke & Zekany  2020 | To describe and categorize patient and nurse perspectives on falls in our unit and to identify nurses’ suggestions  regarding prevention of future falls | - Alarms overwhelmingly the most common prevention strategy suggested  - Nurses did not feel sick, hospitalized patients can be relied on to call for assistance with ambulation  - Using alarms does not immobilize patients - alarm keeps staff aware patients are mobilising and may need help  - Only 1 of 67 patient falls had an activated alarm. The alarm was set to alarm only when the patient was completely out of bed. This fall might have been prevented if a more sensitive setting was activated. A second fall was after the alarm was deactivated by staff frustrated by excessive false alarms from a highly sensitive alarm setting. |
| Hubbartt et al. 2011 | - To determine whether nurses would use an alarm if it were readily accessible  - To obtain feedback about the pilot program and nurses’ experience with and beliefs about using bed alarms for fall prevention | - Ambivalent about alarm effectiveness “Why use the alarms? You have to make routine rounds anyway”  - Only one type of alarm to which nurses must respond. Alarm overload has potential to make alarms ineffective and sensitize nurses against responding. Excessive false alarms  - Times when alarms were effective and a sitter not required  - Alarms worked best with patients who are restless, move quickly  - Agitated patients who constantly attempt to get out of bed need closer observation than alarms can facilitate  - When a fall occurred, investigator automatically asked, “Was a bed alarm in use?” Implied nurses should use alarms  - Hospital had a low fall rate, so nurses believed alarms would not improve patient safety. Nurses unwilling to “give up” another aspect of care to use alarm |
| Iio et al. 2016 | To investigate user evaluations about senior and caregiver intentions to use and feelings of security of a fall detection system in nursing homes  Hypothesis:  A fall detection system using range sensors is accepted by seniors more than commonly used fall detection systems | **Staff issues with current sensor:** False-positive alerts creating alarm fatigue and work disruption; Some seniors avoid the sensors; Malfunctioning alarm  **Intention to use - older person:** 4.30 out of bed sensor; 5.86 fall detection with constant observation**;** 6.04 fall detection without constant observation  **Feelings of security - older person:** 4.69 out of bed sensor; 6.38 fall detection with constant observation; 6.23 fall detection without constant observation  **Intention to use – staff:** 3.66 out of bed sensor**;** 5.78 fall detection with constant observation**;** 5.56 fall detection without constant observation  **Feelings of security – staff:** 4.71 out of bed sensor; 5.85 fall detection with constant observation; 6 fall detection without constant observation  **Older person Sensor preference:**  Out-of-bed sensor not enough, unsafe or unnecessary 12.7%  Out-of-bed sensor is enough and safe 12.7%  Constant monitoring is best 26.4%  Monitoring only for danger situations is best 18.2%  Switching monitoring level depending on situation: 5.5% |
| Jagella et al. 1992 | To examine:  1. Characteristics of residents on alarms  2. Perceived benefits and problems of alarms according to physicians, nurses, aids, administrative staff, and family | - 62% residents had cognitive impairment and unable to ambulate independently  - 54% residents using alarms to eliminate restraints  - 33% residents using alarms due to intrinsic and/or extrinsic factors  - 13% resident reason for alarm not documented  - 70% staff believed alarms effective preventing falls or reduce need for restraint  - 81% family believed alarms an effective alternative to physical restraints |
| Johnston & Magnan 2019 | To evaluate effect of using a new 14-item checklist based on existing hospital-approved fall prevention protocol on nursing staff’s adherence to interventions in change-of-shift handoffs | - Error most frequently observed was incorrect setting of bed alarm  - 90 checklists completed: 19% (n = 17) alarms not set on zone 2  - Staff taught how to recalibrate the bed correctly when all items the patient was likely to have on the bed were on the bed e.g. more than one blanket  - In light of evidence alarms do not prevent falls and may have unintended adverse consequences, the hospital-mandated use of alarms for all at-risk patients at all times is now being challenged. Anecdotally, nurses report bed alarms have, on occasion, “saved the day” in preventing a fall. In view of this deeply held belief, it seems unlikely staff will abandon use of bed alarms completely. However, a more moderate approach to use of bed alarms with zone setting determined by nursing judgment and patient-specific factors would increase nursing autonomy and might benefit some patients by avoiding unintended consequences of bed alarm enforced immobility |
| Jones et al.  2021 | To compare patient perceptions of privacy while toileting with the toilet alarm and with staff at arm’s length  To determine staff perceptions of and level of comfort with toilet alarms as a patient safety measure | **Patient survey:**  - 97% intervention group agreed they were given privacy, 78% in control group  - Intervention group significantly less afraid of falling during hospital than control  - Intervention group felt they had more opportunities for toileting independently  **Staff survey:**  - Lowest score: toilet alarms allowed staff to be more productive: 2.65  - Highest score: impact of toilet alarms on patient safety: 3.06, patient comfort and satisfaction: 3.26  - Alarm easy to use: 71% staff reported “usually” or “always”  - Alarms worked as expected: 54.3% “usually” or “always”; 45.7% “sometimes"  - Patient safe while using toilet alarm: 82.4% "usually" or "always" |
| Kelly et al.  2002 | To measure the effectiveness and operational characteristics of a new device intended to reduce falls in elderly patients at high risk of falling | - Cognitively impaired patients responded well to alarm sound, sitting back down  - 6 patients asked for alarm to be removed or removed it themselves, they resisted limitations on unsupervised mobility  - No adverse skin effects noted when patches were removed after 10 days  - **Staff acceptance** qualitatively appeared high. **Patient acceptance** appeared high. The device largely unnoticed by patients |
| King et al.  2018 | To explore nurses’ experiences with fall prevention in hospital settings and the impact of those  experiences on how nurses provide care to fall risk patients | **Impact of intense fall message on nursing practice:** Fear of falls, resulting investigation and reprimand if falls occurred, job security and public exposure of error  **Protecting:** Nurses protected the unit from being identified a “bad” unit by trying to keep fall number down. Staff stopped whatever they were doing and ran to alarms - often chaos with multiple staff running. The need to run to alarms was reinforced among nurses and a directive from NMs, placing greater stress on nurses and increased anxiety  **Consequences to the nurse:** All nurses stated a fall prevention protocol was not available on their unit. The primary mechanisms communicated to staff to reduce falls was: identify patients at risk; place bed/chair alarms on patients and run to alarms. Nurses were not reducing risks for falls, so efforts to meet hospital goal (zero falls) unsuccessful  **Consequences to the unit:** Over identification of fall risk, “everybody runs” strategy, and continued messaging the unit was not meeting goals, produced low morale |
| King et al.  2021 | To understand older adults' perceptions of and experiences with ambulation during a hospital admission | **Restriction messaging:** Discussions about getting up to walk centered on nurses’ concern for falls  - Messaging about restricted movement delivered through multiple means: orally (by nurses), visually (signs), or auditory (alarms). Most annoying was setting off an alarm. Any movement activated the alarm, resulting in a voice coming over the room intercom telling them to stay put, or having staff rush into their room to tell them to not move. Alarms were very effective in restricting their movement. Many found the alarms offensive and unnecessary |
| Lopez et al. 2010 | To identify constraints in work processes and the work environment (physical, organizational systems and culture, individual, and technical) imposed on acute nurses that may increase the likelihood of patient falls, and to identify workarounds employed by nurses to deal with these constraints | **Workload constraint leads to the need for bed alarms:** Heavy workloads with competing parallel demands and a large proportion of time away from the patient, the task of monitoring falls becomes increasingly difficult. The workaround was to use alarms, but these were employed inconsistently and success varied. Alarms were either too sensitive or too insensitive, non-intuitive to use, inaudible at distances, often deactivated and not reactivated, and defective sometimes  **Limitations in the physical environment constraint lead to informal surveillance:**  Nurses spent only 33% of time in patient rooms, divided among 4-5 patients. No means of formal surveillance to deal with this constraint. From the nursing station there was a direct viewing angle into only 3 of 17 patient rooms. The workaround was to add informal video and audio surveillance to improve patient visibility. Redesign strategies included relocating nurses’ indirect care tasks within close physical proximity to bedside and allow more space for family members to stay overnight |
| Matsuo et al. 2008 | To determine the benefits of using the vigilance monitoring system | - 76% supported the usefulness/effectiveness of the vigilance system  - Patient satisfaction in response to call lights and overall nursing care improved  - Following implementation, staff experienced alarm fatigue. Everyone began to ignore the alarms. Despite reeducation and immediate improvement in patient outcomes, nurses continue to feel burdened with multiple alarms. However, they see the value of increased vigilance that results from the alarms, and the purpose of implementing the monitoring device was to support nursing staff by providing a tool to improve patient surveillance |
| Mitchell et al. 2018 | What activities are hospitals undertaking to prevent falls and harm from falls, how much does this cost? | - Alarms were used at each health service and constituted approx. 11% of total falls prevention spend or AU $909 per bed per year  - Costs included alarm hire or purchase and ward staff time spent sourcing, setting up and responding to alarms on the ward  Health Service A (private) restricted use of falls alarms to subacute wards |
| Okumoto et al.  2020 | - To express nurses’ perceptions of the bed alarm system from the viewpoint of considering it as a form of physical restraint  - To identify the factors related to these perceptions | - 214 (74.0%) nurses considered bed alarm a form of physical restraint  - Perceptions were associated with the hospitals they belonged to  - Education did not affect nurses’ perception, irrespective of whether they completed ethical, physical, or bed alarm education  - Newly graduated nurses were 4.1 times likelier than expert nurses to consider alarm systems a restraint |
| Porter et al.  2018 | To address the knowledge gap between implementing and sustaining evidence based fall prevention practices | **Communication of fall risk and fall prevention strategies:** NM provided daily reminder to staff to set bed alarms**.** The unit clerk linked alarms to both call light system and in-house smartphone, so “everyone” was alerted to a patient’s movement. Unclear whether “everyone” included non-nurses  **Nurse to patient staffing levels:** Staffing levels and alarm usage directly affected ability to prevent some falls. Difficulties attending to simultaneous patient needs  **Variations in unit-specific fall prevention strategies** |
| Pu et al.  2026 | To investigate whether “reduced” or “eliminated” mobilisation alarm conditions are non-inferior to “current” practice on wards with high levels of mobilisation alarm use for the prevention of falls | Baseline alarm rates of 18 wards ranged from 3.29% to 24.35%  Reduction in use of alarms is not inferior to usual care, but uncertainty remains around complete elimination  High use of alarms did not lead to significantly lower fall rates compared to reduced or eliminated use |
| Radecki et al. 2018 | To describe the patient's perspective of fall prevention in an acute care setting to aid in the design of patient centered strategies | **What I see and hear:** “Well, they [nurses] got my bed alarm, chair alarm. Whenever I move or stand up, they're in here.”  **Usefulness of equipment:**  Many found interventions, such as the alarm and gait belt, useful for nursing. Some felt alarms were restrictive, others described alarms did not deter intentions to get up unassisted. Only 1 patient described the alarm as a useful tool to reminder to stay in bed/call for help. Intentionally using the alarm as a call light to ensure a rapid response could contribute to alarm fatigue |
| Sarkies et al.  2019 | To evaluate effectiveness of video vs. written knowledge translation strategies … for improving … knowledge of evidence provided in a scientific journal article across 3 health contexts | Generally there was a low level of correct responses to the knowledge test for each health context - alarms lowest out of 3 groups (education, DVT) Control: 6/44 (14%); Written: 8/38 (21%); Video: 15/37 (39%) |
| Shever et al.  2011 | To describe nursing practices around fall prevention, as perceived by nurse managers, in adult, medical-surgical nursing units | **Most common interventions:**  - Alarms (90%), rounds (70%), sitters (68%), relocating patient closer to nurses’ station (56%)  - 29% NUMs identified physical restraints as an intervention to prevent falls 10% mentioned ambulation  - 6% NUMs stated it was standard for all patients to have alarm activated. Fall risk was most frequently (44%) identified reason for alarm activation, then specific risk factors (confused, agitated, heavily medicated; 28%), nursing judgment (16%) and others (12%) |
| Skolka et al. 2023 | To reduce unwitnessed inpatient falls on the neurology services floor by 20% over 15 months | **Confident in ability to operate bed alarms:** Pre: 4.5 Post: 4.8 **chair alarms:** Pre: 4.1 Post: 4.7  **Before leaving a patient’s room, assess whether bed/chair alarm is on?** Pre: 4.2 Post: 4.6  **Education on operating alarms will improve (or improved) my ability to operate the bed and chair alarms:** Pre: 3.6 Post: 4.4 |
| Staggs et al. 2020 | To examine the prevalence of and unit-level variation in alarm use and estimate the amount of variability in alarm use associated with patient- versus unit-level factors | - Alarms in use for 531 (36%) patients  **Patient characteristics associated with higher odds of alarm use:** recent fall, ambulation assistance, poor mobility judgment, and altered mental status. Presence of a sitter was the strongest correlate associated with substantially lower odds of alarm  - Unit assignment a stronger predictor of alarm use than patient-level variables  - The best predictive performance was achieved where both unit assignment and patient information included as predictors |
| Stephen & Campbell  2023 | To explore how older adults with cognitive impairment perceive and experience falls prevention alarms in hospital | **Communication and collaboration with staff:** Alarms ‘extra back up’, awareness alarms are just one aspect of falls prevention. All were aware an alarm was in use, despite not provided with consistent information about it. Alarms required due to personal factors (dizziness and delusions). Some erroneously thought alarms were provided for other purposes. Some thought they did not require an alarm but felt obliged to adhere to staff recommendations. Some requested or removed alarm. Occasionally, preference was over-ridden; other times alarm use negotiated  **Rationalisation of alarm use:** Reconsideration of role of alarm was not documented post falls**.** Alarms introduced as routine practice but lacked individualization and follow-up  - Negative experiences: alarm hypersensitivity, sleep disturbance, perceived negative nurse reactions, physical discomfort, and restraint  - Alarms were switched on and positioned correctly 66% of observations  - Some perceived alarms useful if staff could respond promptly. Others ambivalent - useful for others, rather than themselves. Some thought falls were unavoidable |
| Stephen et al.  2025 | To explore whether person- centred principles are applied to:  Why staff use alarms  How staff use alarms | **Understaffed, Under Skilled, Under Pressure:** Insufficient staff for increased patient complexity**.** Alarms useful overnight due to reduced staffing. Alarms also believed ineffective because not enough staff to answer them promptly**.** Staff responsible for multiple alarms. Stressed with the impossible task of responding to simultaneous alarms or leaving a critical task or high risk patient. Most staff never experienced working without the option of alarms. Busy clinical demands and lack of supervision meant alarms were used on default. More experienced nurses/allied health more confident alarms were not essential  **Alarm Impact on Stress and Workload:** Staff petrified patients will fall, and wanted to implement all available strategies**.** Without alarms, there was a sense staff would burn out with worry patients would fall, alarms offered a reprieve from constant vigilance**.** Staff negotiated conflicts within the team of who should and should not use alarms, when they should cease and which staff were or were not responding. Staff were annoyed by frequent false alarms and frustrated alarms took away from other important tasks. Staff worried if the alarm was turned on, positioned correctly, working properly or who was available to answer it  **Negotiating Patient Safety and Patient Preference:** Staff consistently described patients disliked alarms, but felt compelled to use them. Patient choice often overridden, but there were instances where alarms were removed based on patient discomfort**.** Including patients with cognitive impairment in alarm decisions was difficult, so alarms often introduced without consent  **Engaging Family as a Resource:** Implementing alarms was quicker than proactively engaging family for individualised interventions. Limited experience and confidence, lack of formal family engagement processes and impracticality of family input overnight triggered alarm use**.** Staff felt ill-equipped to justify non-use of alarms, so continued using them  **Sharing Responsibility for Alarms and Falls Prevention:** No formal procedures who implements or removes alarms**.** Alarm response varied from nursing team versus individual nurse**.** Allied health and nurses shared different views on usefulness of alarms and what allied health's role should be with alarms. Nurses could overrule allied health decisions  **Navigating Ambiguity and Fearing Consequences:** Fall guidelines or risk management tools listed alarms as an option. Many interpreted this as mandating, rather than suggesting, alarms**.** Little faith their organisation would support them in defending their professional reputation if patients fell and an alarm not used. Team alarm removal decisions were valued to share liability |
| Subermaniam et al.  2017 | - To determine the effectiveness of the device in alerting nurses to bed-exit episodes  - To determine the effect of the introduction of the device on ward nurses’ workload, and nurses’ perception on usefulness of device preventing falls | **Workload:** Overall reduction in nurses’ workload with introduction of alarm. Significant reduction in mental and physical demand  **Falls detection:** Helped me to detect falls fast: Agreed: 25; Disagreed/not sure: 5  Able to alert me accurately in regards to patients’ movement: Agreed: 15; Disagreed/not sure: 15  **Workload:** Helped reduce my work load: Agreed: 17; Disagreed/not sure: 13  **Usage of the bed alarm:** Simple technology and easy to operate/handle: Agreed: 29 Disagreed/not sure: 1  **Bed alarm use in future:** Will use the bed alarm in future for my patient: Agreed: 26; Disagreed/not sure: 4  It is suitable to be used for my elderly patients: Agreed: 25; Disagreed/not sure: 5 |
| Takase 2023 | To explore, from the perspectives of nurses, how patients, the environment, nurses interact with each other to contribute to patient falls | - 16 major fall clusters extracted. Unproductive use of bed alarm formed 1 cluster. 202 reports (4.84% of total reports) formed alarm cluster  - Nurses did not check if the alarm was activated and set to the right mode, so alarm did not work as expected. Some patients avoided activating sensor by stepping over it. Even if alarm went off, nurses could not respond to the patient’s needs before patient moved |
| Tideiksaar et al.  1993 | To determine the efficacy of an alarm system to reduce the number of bed falls in an acute-care hospital setting | - Alarm not associated with bodily harm or interference with care  - Patients, family and nursing staff universally approved of alarms. Preferable to using mechanical restraints  - Nurses found the system user friendly, easy to install and operate, remaining trouble free. False alarms (16%) were the only factor that frustrated nurses and inhibited use. Alarm removed from one patient and replaced with mechanical restraints  - Nurses felt they had sufficient time to respond to avoid falls |
| Timmons et al.  2019 | - To critique the assumptions about how RCTs work and to analyse the ‘failure’ of a patient safety intervention  - To examine the fall and its prevention as a situated phenomenon of knowledge that is made and unmade through intra-actions between environment, culture, humans and technologies | **Staff:** RNs tended not to carry the pager, claiming ‘the trained staff have got duties to do that they can’t be disturbed. Pagers often carried by HCAs suggesting it was not a priority by RNs  - Staff complained alarms were unreliable, faulty and poor battery life on pagers  - Ignored alarms from patients staff ‘knew’ to be bed-bound, or ‘healthy’ or because they were busy with another patient  - Occasionally alarm turned off due to not enough staff to answer it  - Debate about the 8 second delay between sensor activation, and alarm going off. It was both too long and not long enough  - Critical views were shared by most, but not universal. Some were positive  **Patients:** A number of patients were unaware of the sensor. Patients talked little about it  - Very limited nurse-initiated interaction with patient on resetting the sensor alert  - Patients sometimes limited their movement to avoid activating sensor. Some asked to have it removed or disconnected it  - Some felt reassured having the sensor  - Some in the non-intervention group were grateful they did not to require one  - Staff claimed some patients deliberately set the alarm off to get attention |
| Torres et al.  2017 | - To explore acceptability of the system to inpatients  - To explore performance of wearable sensor based system in identifying movement transitions of bed and chair exits in hospitalized older people | **Improved perception of alarm after participating in trial**  - Pre-trial: Overall average score: 7.83  - Post-trial: Overall average score: 9.03  - Overall average score of 8.99 for the Sensor Acceptance Model (physical activity, anxiety, equipment and privacy). Lowest score was for "I just forgot I am wearing it" demonstrating an overall concern for the high visibility of the sensor during the trials |
| Turner et al.  2025 | - To identify, conceptualise and select strategies to deimplement fall prevention alarms  - To obtain feedback from key stakeholders on tailoring selected deimplementation strategies for the local hospital context | **Group concept mapping:** 79 statements grouped into 9 clusters responsive to focus prompt: ‘to safely reduce bed/chair alarms for fall prevention in our hospital, we need’: Pharmacy and Medication Oversight; Environmental Enhancements; Tailored Resources and Supplies; Surveillance and Visibility; Appropriate Staffing and Team Composition; Hospital Culture for Fall Prevention; Appropriate Policies and Procedures; Education; Communication strategies  **Item-level ratings above average on importance and below average on current use:**  - having mobility technician and specially trained unlicensed staff (eg, volunteers) to assist with fall prevention efforts  - helping staff/leadership move away from idea alarms are a universal strategy  - tailor interventions to specific patient risk factors not risk score  **Barriers to deimplementation:** leadership, lack of knowledge about evidence around alarms, family members’ expectations alarms should always be on fall risk patients, use of universal rather than patient-specific policies. Many leaders and staff aware of downsides of overusing alarms (alarm fatigue, patient mobility restriction) but lack awareness of research demonstrating alarms are not effective. Insufficient staffing and fidelity monitoring procedures to reliably implement evidence-based fall prevention interventions  **Implementation package:** Included theory-based strategies recommended by the research team (external facilitation, education, champions, audit feedback) and stakeholder-driven strategies based on GCM / focus groups. Some sites implemented additional strategies i.e. redefining or creating new roles such as mobility technician (n=4), revising policies / procedures to promote tailoring fall prevention interventions based on patient risk factors rather than risk score (n=5) and improving falls risk medication review (n=3) |
| Tzeng & Yin  2015 | To identify **perceived** top 10 highly effective interventions to prevent fall injuries of inpatients based on perceptions of RN staff by specialty area in acute hospitals | - 560/2170 RNs completed survey. Response rate 25.81%  - Alarms only rated in the top 10 perceived effective and frequently used interventions in one of the 10 units  - Perceived effectiveness mean 4.12/5 and frequency of use 4.06/5  - Based on the ranks, the perceived top 3 effective interventions did not match the 3 most frequently used interventions |
| Vandenberg et al.  2017 | To examine how existing fall prevention technology was experienced within nursing home nurses’ environment and workflow | **U.S: “Everybody’s supposed to be listening” Falls a problem:** Staff cannot coordinate care. May hesitate, unaware where alarm is alerting from. Chaos as staff answered alarms and left other residents unattended. Staff unaware who was taking care of the problem until arriving on scene**.** Alarms faulty, broke, or easy to dismantle by residents, alerted too late to prevent falls, and emitted a piercing noise that inhibited movement, caused agitation and haunted staff. More staffing wanted  **NL: “I just feel normal, during night shift too” Rarely encountered falls**: Relaxed attitude towards falling from management. Problem of falling “less important than taking people’s freedom away.” Safety culture of individualized care with delegated and coordinated responsibilities enabled by technology and care unit configuration. Make agreements (recorded in care plans) with residents about tradeoff between safety and freedom. NL staff use a mobile infrared sensor to detect movement. Sensor positioned differently depending on resident’s fall risk and movement speed: on a bed’s headboard (aimed at rising torso), on side of bed aimed at limbs extending over edge of bed), on floor (showing feet touching ground), or at door (indicating exit from the room). Alarms are directed silently to assigned nurses’ mobile. If unit staff do not respond, staff of second closest unit are alerted, and if they do not respond, staff at multiple units are alerted. Nurse can also call and talk to resident |
| Vassallo et al.  2005 | To explore attitudes to restraint and what are acceptable fall prevention measures in hospital | **Consider bed/chair alarms a restraint:**  **Patients/relative:** Disagree: 60**;** Not sure: 10**;** Agree: 30  **Staff:** Disagree: 67; Not sure: 12; Agree: 21  - 25.5% agreement bed/chair alarms perceived as restraint. 80% agreement bed/chair alarms are acceptable |
| Wen 2022 | - To describe the implementation and experience of an innovative SPCS (Smart Patient Care System)  - To describe nurses’ user experience of SPCS  - To compare the perception of the alarm handling process, response time, false alarm rates between SPCS and TPCS | - The immediate communication via phone is helpful: Strongly agree (52%) 3.92  - The three‑stage bed‑exit alert is helpful: Neutral (40%) Agree (28%) 3.68  - Care efficiency is improved: Agree (64%) 3.60  - Awareness of patients’ in‑time situation is increased: Neutral (48%) Agree (52%) 3.52  - Care quality is increased: Neutral (48%) Agree (48%) 3.44  - The event presentation is easy to understand: Neutral (32%) Agree (56%) 3.44  - The interface is easy to use: Neutral (56%) Agree (44%) 3.44  - Stress caused by caring for patients is reduced: Neutral (60%) Agree (28%) 3.12 |
| Widder 1985 | To test effectiveness of Ambularm on reducing fall rates | - Explanation of the function and purpose of the device was essential in gaining patient cooperation. No patients experienced any problems. A few confused patients tried to remove it. The manufacturer is changing the way the device attaches to make removal less easy  - Some patients found it difficult to use existing communication systems because of confusion, speech difficulty, or impaired vision. Appeared to feel more secure with Ambularm as a passive method of summoning help  - Nurses enthusiastic, quickly relied on it. Not a substitute for frequent checks or restraints, but it decreased need for restraints.  - Sometimes the alarm alert caused patients to cease effort to stand  - Ambularms used often ≥6 usually in use on the 32-bed general medical unit |
| Wolf et al.  2013 | To show whether application of a technical system detects patients getting up translates into a reduction of falls on a geriatric ward | - A patient reported the sensor did not bother her at all, did not feel constricted  - Nurses open and interested in the technology  - Nurses confirmed alarm reliably detected attempts to get up, few false alerts |
| Wong Shee et al.  2014 | To evaluate the effectiveness of an electronic sensor exit alarm system on fall incidence and fall-related injury rates in a subacute hospital ward for patients with cognitive impairment and high falls risk  To evaluate acceptability of the system for nurses | - 91.7% thought alarms useful for monitoring patients getting out of bed / chair. 91.6% satisfied with the system  **Limitations of the alarm system:** Sensor mat too narrow and needed to be positioned in exactly right position. Staff forget to turn alarms on. Routing alarms through the nurse call system was a problem - unable to distinguish from call bell  **Staff identified 2 main areas for potential patient harm:** Sensor mat connector bump a pressure risk; Alarm cords a trip hazard  **Safety benefits compared with existing floor sensor mats:** Floor mats difficult to push equipment (hoists, wheelchairs) over and put staff at injury risk**.** The system was versatile between bed and chair. Sensor mats could be rolled up for storage, unlike floor sensor mats  - Education on using alarm not consistently provided to casual or new ward staff which was a barrier to optimal alarm use |
| Yesmin et al. 2022 | To explore an advanced IoT (Internet of Things) -based intervention in a hospital setting and demonstrate its impact on patient safety | - RNs largely agreed smart beds helped to significantly reduce falls  - Despite all of the advantages, nurses also noted complexities. E.g. beds frequently became unplugged while delivering care; they initially needed training on reconnecting and adjusting all of the bed’s many parameters |
| Zwijsen et al.  2011 | To explore how care professionals and family members of RAC residents with dementia in the Netherlands experience and define the concept of restraint | - Over half did not regard surveillance technology devices as restraints  - Movement sensor (and bed rail) scored the least in moral distress scale. Safety belt, forced medication, acoustic monitoring, geriatric chair/table top, camouflaged medication, chips in clothing all scored higher  **Criteria used to define an intervention as a restraint:**  The perception of the resident: If a resident does not notice an intervention, it cannot be regarded as a restraint; Advancing dementia complicated interpretation of resident - problematic when resident’s perception is used to define restraining qualities of interventions  The intention of the care professional: The intention is not to restrict the resident, but to protect from harm or give them help they need and therefore not labeled as restraints  Invasion of privacy: with surveillance as restraint, an extra criteria was used that was not applied to other types of restraints - privacy  - Relatives did not regard monitoring devices as restraint. Surprised others did  Differences between respondents: Nurses and relatives felt in some situations there was no choice but to use restraint. However, key persons, who were mostly RACF doctors, did not always feel restraints should be used |
| Zwijsen et al.  2012 | To examine the views of professionals in dementia care on the use of surveillance technology as an alternative to physical restraints.  How is surveillance technology currently being used in Dutch nursing homes?  What are, according to the professionals, the pros and cons of using surveillance technology in nursing home care? | **Providing additional safety:** Often use combination of restraint and surveillance device e.g. sensor next to bed with rails to know if resident climbs over rail, prevent long lies  - Provides a feeling of extra safety. Tempting to use surveillance on almost all residents. Staff did not regard surveillance technology as harmful. Felt they should be able to place devices whenever they felt it could give them or others more piece of mind about resident safety  **Providing more freedom for residents:** Prevents need to disturb residents’ sleep or daily activities. Nurses can rely on the devices to tell them when to act  **Limitations of surveillance technology:** Surveillance technology cannot guarantee quick help. Caregivers do not have the capacity to react to all alerts. It helps when staff know residents well to prioritise response  - Devices do not always work properly. Nurses do extra rounds to ensure all devices are working correctly  - Surveillance could violate privacy: Some who worked in homes that used a lot of surveillance expressed privacy concerns but this had not led to action to reduce use  - Participants did not raise concerns previously raised in research e.g. impact quality of care relationship by reducing moments of face to face contact - cared 'from a distance', loss of dignity through stigmatisation |
